# Histone depletion prevents telomere fusions in pre-senescent cells

**DOI:** 10.1371/journal.pgen.1007407

**Published:** 2018-06-07

**Authors:** Marta Barrientos-Moreno, Marina Murillo-Pineda, Ana M. Muñoz-Cabello, Félix Prado

**Affiliations:** Department of Genome Biology, Andalusian Molecular Biology and Regenerative Medicine Center (CABIMER), CSIC-University of Seville-University Pablo de Olavide, Seville, Spain; Chinese Academy of Sciences, CHINA

## Abstract

Upon telomerase inactivation, telomeres gradually shorten with each cell division until cells enter replicative senescence. In *Saccharomyces cerevisiae*, the kinases Mec1/ATR and Tel1/ATM protect the genome during pre-senescence by preventing telomere-telomere fusions (T-TFs) and the subsequent genetic instability associated with fusion-bridge-breakage cycles. Here we report that T-TFs in *mec1Δ tel1Δ* cells can be suppressed by reducing the pool of available histones. This protection associates neither with changes in bulk telomere length nor with major changes in the structure of subtelomeric chromatin. We show that the absence of Mec1 and Tel1 strongly augments double-strand break (DSB) repair by non-homologous end joining (NHEJ), which might contribute to the high frequency of T-TFs in *mec1Δ tel1Δ* cells. However, histone depletion does not prevent telomere fusions by inhibiting NHEJ, which is actually increased in histone-depleted cells. Rather, histone depletion protects telomeres from fusions by homologous recombination (HR), even though HR is proficient in maintaining the proliferative state of pre-senescent *mec1Δ tel1Δ* cells. Therefore, HR during pre-senescence not only helps stalled replication forks but also prevents T-TFs by a mechanism that, in contrast to the previous one, is promoted by a reduction in the histone pool and can occur in the absence of Rad51. Our results further suggest that the Mec1-dependent depletion of histones that occurs during pre-senescence in cells without telomerase (*tlc1Δ*) prevents T-TFs by favoring the processing of unprotected telomeres by Rad51-independent HR.

## Author summary

Telomere shortening upon telomerase inactivation leads to an irreversible cell division arrest known as replicative senescence, which is considered as a tumor suppressor mechanism. Since pre-senescence is critical for tissue homeostasis, cells are endowed with recombination mechanisms that facilitate the replication of short telomeres and prevent premature entry into senescence. Consequently, pre-senescent cells divide with critically short telomeres, which have lost most of their shelterin proteins. The tumor suppressor genes ATR and ATM, as well as their yeast homologs Mec1 and Tel1, prevent telomere fusions during pre-senescence by unknown mechanisms. Here we show that the absence of Mec1 and Tel1 strongly augments DSB repair by non-homologous end joining, which might explain the high rate of telomere fusions in *mec1Δ tel1Δ* cells. Moreover, we show that a reduction in the pool of available histones prevents telomere fusions in *mec1Δ tel1Δ* cells by stimulating Rad51-independent homologous recombination. Our results suggest that the Mec1-dependent process of histone depletion that accompanies pre-senescence in cells lacking telomerase activity is required to prevent telomere fusions by promoting the processing of unprotected telomeres by recombination instead of non-homologous end joining.

## Introduction

Telomeres are highly specialized nucleoprotein structures that hide the ends of chromosomes from double-strand break (DSB) repair and DNA damage checkpoint activities. In this way, telomeres protect the chromosome ends from fusions and degradations and from eliciting an erroneous DNA damage response. Accordingly, defects in telomere maintenance are linked to cancer and aging [1]. Telomere DNA consists of repeated DNA sequences that end in a 3′ single-stranded G-rich tail. To compensate for the natural shortening that telomeres undergo every cell cycle during DNA replication, many cells express a reverse transcriptase, the telomerase, which adds telomere repeats. However, telomerase expression is repressed in many tissues of multicellular organisms, leading to continuous telomere erosion that eventually activates replicative senescence [2]. Premature senescence entry can affect tissue homeostasis [3] and accordingly, cells are endowed with mechanisms to maintain the proliferative state of cells with short telomeres. As a major risk for genome integrity during this time is the instability of critically short telomeres, an essential task during pre-senescence is to protect these telomeres. In sharp contrast to dysfunctional telomeres caused by mutations in the telomeric shelterin proteins, eroded telomeres by physiological shortening are able to repress non-homologous end joining (NHEJ), thus preventing telomere-telomere fusions (T-TFs) and the subsequent genetic instability associated with fusion-bridge-breakage cycles [4,5].

In yeast, telomeres consist of ~300 bp of TG_1-3_ repeats that are covered by 15–20 molecules of Rap1 (repressor activator protein 1) and its partners Rif1 and Rif2, and ~12–15 bases of a G tail that are covered by the Cdc13/Stn1/Ten1 complex. Similar to most organisms, yeast also contains two classes of subtelomeric elements: X elements, which are present at virtually all telomeres, and Y′-elements, which are present in zero to four tandem copies immediately internal to the telomere repeats [6]. In yeast cells that lack telomerase, telomeres progressively shorten with each cell cycle until cells enter replicative senescence with critically short but protected telomeres [7–10].

Mec1 and Tel1 (yeast homologs of the tumor suppressor genes ATR and ATM, respectively) are master checkpoint kinases with specific and redundant roles in many processes related to genome integrity, such as DSB signaling [11,12]. Specifically, Mec1 transduces the signal that activates senescence in cells lacking telomerase when telomeres reach a critical length [13,14]. Telomeres regulate the binding and activity of many DNA repair and checkpoint factors that are essential for telomere maintenance but prevent that these factors process the chromosome ends as DSBs. Thus, Mec1/ATR and Tel1/ATM binding to telomeres is regulated in order to facilitate their role in promoting the recruitment of the telomerase to short telomeres [15–19] without leading to an inadvertent activation of the DNA damage checkpoint signaling [20–22]. The telomerase recruitment function is carried out mainly by Tel1 in wild-type cells, although Mec1 can partially complement this function in the absence of Tel1 [17]. Consistently, telomeres are barely affected in *mec1Δ* cells, are very short but stable in *tel1Δ* cells, and only the lack of both Mec1 and Tel1 leads to short and unstable telomeres and to the activation of replicative senescence [7,8]. However, and in contrast to cells lacking telomerase, *mec1Δ tel1Δ* cells accumulate T-TFs, indicating that Mec1 and Tel1 have additional functions in protecting telomeres [9,23,24]. These functions seem to be conserved as inferred from the accumulation of telomere fusions observed in yeast, fly and mammalian cells lacking ATM and ATR [25–28].

Yeast and mammalian cells also share a reduction in the synthesis of histones during pre-senescence [29,30]. The mechanism of histone reduction has been elucidated in yeast cells lacking the telomerase RNA coding gene (*TLC1*) [30]. In particular, it has been reported that telomere shortening is accompanied by a relocalization of Rap1 from eroded telomeres to the promoter of hundreds of new genes. A subset of these genes includes the core histone–encoding genes, which are repressed by Rap1 leading to a reduction in the pool of available histones and a loss of histones at Rap1-targeted promoters. Importantly, Rap1 relocalization and histone depletion require Mec1 [30].

In this study, we asked what defects in *mec1Δ tel1Δ* cells are due to their inability to reduce the level of histones as compared to *tlc1Δ* cells. We show that an induced reduction in the pool of available histones in *mec1Δ tel1Δ* does not affect the length of telomeres or the entry into senescence, but prevents T-TFs. This histone depletion–induced protection require a HR mechanism that, in contrast to the one that maintains the proliferative state of *mec1Δ tel1Δ* cells during pre-senescence, can occur in the absence of Rad51. Likewise, cells lacking Tlc1 requires Rad51-independent HR to prevent telomere fusions, opening the possibility that the Mec1-dependent depletion of histones that occurs during pre-senescence protects telomeres from fusions by favoring the repair of unprotected ends by HR rather than NHEJ.

## Results

### Histone depletion induction prevents T-TFs in *mec1Δ tel1Δ* pre-senescent cells

**Fig 1:**
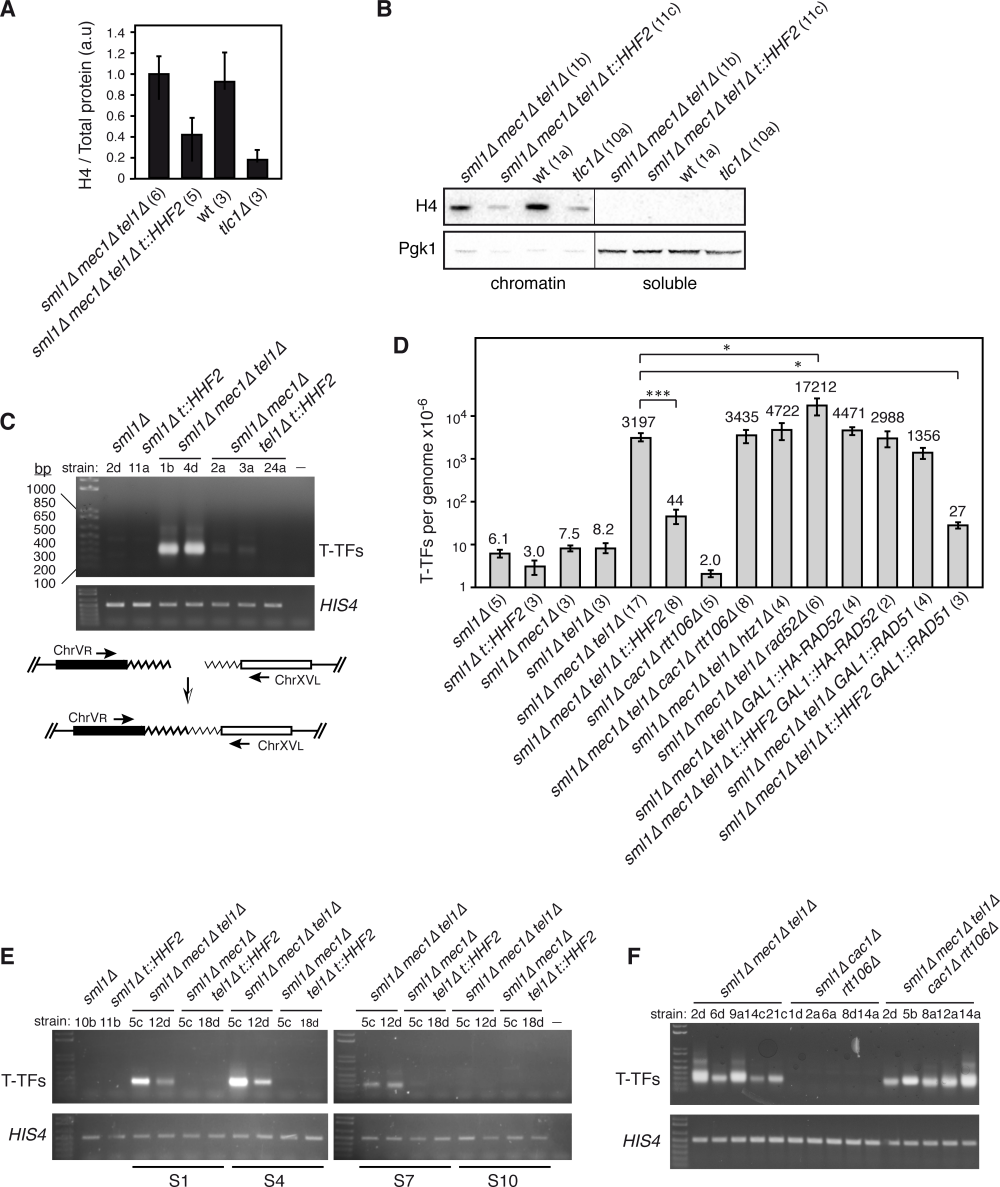
Histone depletion prevents T-TFs in *mec1Δ tel1Δ* pre-senescent cells. **(A)** Histone H4 levels in *mec1Δ tel1Δ* and *mec1Δ tel1Δ t*::*HHF2* cells (*sml1Δ* background), and wild type and *tlc1Δ* cells from streak 1-derived cultures as determined by western blot. The amount of histone H4 was normalized to the total amount of protein. The average and SEM of the indicated number of independent strains are shown. **(B)** Histone H4 and Pgk1 levels at chromatin and soluble fractions of the indicated strains from S1-derived cultures. **(C)** T-TFs in wild type, *t*::*HHF2*, *mec1Δ tel1Δ* and *mec1Δ tel1Δ t*::*HHF2* (*sml1Δ* background) from liquid cultures from streak 1 as determined by semiquantitative PCR. A PCR control without DNA was included (-). A DNA fragment from *HIS4* was PCR-amplified as input control. A scheme of the PCR assay to follow the accumulation of T-TFs is shown. **(D)** T-TF frequency of the indicated strains as determined by quantitative PCR from S1 streaks. The average and SEM are shown, except for *mec1Δ tel1Δ t*::*HHF2 GAL1*::*HA-RAD52* in which the range is indicated, as n = 2. The number of DNA samples from independent strains, which include those used for semiquantitative PCR and telomere length analyses, is shown in parenthesis for each genotype. One (*P*-values <0.01) and three (*P*-values *<*0.0001) asterisks indicate statistically significant difference according to a Student’s *t*-test (Mann-Whitney). **(E)** Accumulation of T-TFs during pre-senescence (streak S1), senescence (S4), and post-senescence (S7 and S10). **(F)** T-TFs in *mec1Δ tel1Δ*, *cac1Δ rtt106Δ* and *mec1Δ tel1Δ*, *cac1Δ rtt106Δ* (*sml1Δ* background) from streak 1 biomass as determined by semiquantitative PCR. All analyses were performed with haploid strains (indicated below each genotype) obtained from heterozygous diploids and streaked for several times on rich medium plates.

To address the genetic consequences of the inability to reduce the amount of histones during pre-senescence of cells lacking Mec1 and Tel1, we induced a partial depletion of histones in *mec1Δ tel1Δ* cells using a previously reported genetic system, in which the sole source of histone H4 is under the control of the doxycycline-inducible *tet* promoter (*t*::*HHF2*) [31]. For this, *MEC1/mec1Δ TEL1/tel1Δ HHF1/hhf1Δ HHF2/hhf2Δ* diploids transformed with the plasmid p413TARtetH4 were dissected on plates containing rich medium with 5 μg/mL doxycycline and the colonies were streaked on the same medium; a smear of cells from this streak was then restreaked after 3 days, and this step was repeated for several times (each streak involved ~20 generations). Mec1 is essential to maintain the levels of dNTPs during replication and DNA damage, but can be eliminated without affecting viability in cells lacking Sml1, a Mec1-regulated inhibitor of the dNTPs synthesis [32]. Since the intracellular pool of dNTPs has a direct impact on telomere biology [33–35], all analyses were performed in *sml1Δ* strains except for *tlc1Δ*, which was compared with its isogenic wild-type strain. Wild-type and *mec1Δ tel1Δ* cells from streak 1 displayed similar levels of histone H4, which dropped two-fold in *mec1Δ tel1Δ t*::*HHF2* cells (Fig 1A). However, histone levels in *mec1Δ tel1Δ t*::*HHF2* cells were still higher than those displayed by cells lacking telomerase. This difference was confirmed by chromatin fractionation and western blot (Fig 1B and S1A Fig).

To follow senescence, we analyzed cell growth during several streaks from different spores of each genotype. As previously reported, cells lacking Mec1 and Tel1 senesced after 3 to 5 streaks but a small fraction of them survived senescence (S1B and S1C Fig) [7]. This loss of cell viability was less clear in some clones, likely due to the high cell-to-cell heterogeneity in the entry into senescence [36]. Moreover, viability was much lower in *mec1Δ tel1Δ* cells than in *tlc1Δ* cells (S1C Fig), consistent with the major functions of Mec1 and Tel1 in genome integrity. Parallel cultures from *mec1Δ tel1Δ t*::*HHF2* spores from the same diploids also displayed high growth clonal variation with a similar pattern of senescence (S1B Fig) and viability (S1C Fig). However, a more detailed analysis showed that histone depletion slightly rescued the growth defects of *mec1Δ tel1Δ* pre-senescent cells (S1D Fig), and this suppression was not associated with an increase in cell viability but with a slight reduction in the doubling time (S1E and S1F Fig).

To address whether histone depletion prevents T-TFs in cells lacking Mec1 and Tel1, DNA from liquid cultures from streak 1 was isolated and amplified by semiquantitative PCR to specifically detect T-TFs between chromosomes V and XV as previously reported [9]. Histone depletion largely suppressed T-TFs in *mec1Δ tel1Δ t*::*HHF2* pre-senescent cells (Fig 1C). To calculate more precisely this effect, the frequency of T-TFs was determined by quantitative PCR (Fig 1D). The frequency of telomere fusions between the right arm of chromosome V and the left arm of chromosome XV was 3.2 × 10^−3^/genome in *mec1Δ tel1Δ* cells. Histone depletion suppressed T-TFs in *mec1Δ tel1Δ t*::*HHF2* an average of ~73 times. Finally, a detailed analysis in pre-senescent (streak S1), senescent (streak S4) and surviving cells (streaks S7 and S10) showed that T-TFs accumulated in *mec1Δ tel1Δ* during pre-senescence but disappeared in the surviving cells, and that histone depletion prevented T-TFs during the whole process (Fig 1E). Indeed, T-TF accumulation in *mec1Δ tel1Δ* and protection by histone depletion was observed as early as in cultures inoculated directly from the spore (~25–30 generations) (S2A Fig).

Many of the phenotypes associated with partial depletion of histone H4 can be mimicked in *cac1Δ rtt106Δ* replication-coupled chromatin assembly mutants: loss of chromatin integrity and negative supercoiling, replication fork instability, hyper-recombination and chromosome missegregation [37–41]. Thus, we analyzed T-TFs in *mec1Δ tel1Δ cac1Δ rtt106Δ* cells from streak S1 to test whether telomere protection in *mec1Δ tel1Δ t*::*HHF2* was due to global defects in chromatin assembly. Notably, the absence of Cac1 and Rtt106 did not reduce the amount of T-TFs induced by *mec1Δ tel1Δ* (Fig 1D and 1F), suggesting that the effects of histone depletion in preventing T-TFs are not due to global defects in replication-coupled chromatin assembly.

In *Drosophila melanogaster*, the accumulation of T-TFs in the absence of ATR and ATM can be suppressed by depleting the histone variant H2A.Z, which restores the loading of the HOAP capping protein [42]. Given that histone depletion is likely to reduce the amount of H2A.Z (Htz1 in yeast)-containing nucleosomes, we addressed the effect of the lack of Htz1 in the formation of T-TFs in *mec1Δ tel1Δ* cells. The absence of Htz1 did not prevent the formation of T-TFs in *mec1Δ tel1Δ htz1Δ* (Fig 1D and S2B Fig). Interestingly, both *mec1Δ tel1Δ cac1Δ rtt106Δ* and *mec1Δ tel1Δ htz1Δ* cells senesced much earlier (streaks S2-S3) than *mec1Δ tel1Δ* cells, and surviving cells took longer to appear (S2C and S2D Fig), suggesting that Cac1 and Rtt106, as well as Htz1, are required to delay the entry into senescence.

### Telomere protection against fusions by histone depletion in *mec1Δ tel1Δ t*::*HHF2* is not associated with bulk telomere elongation

**Fig 2:**
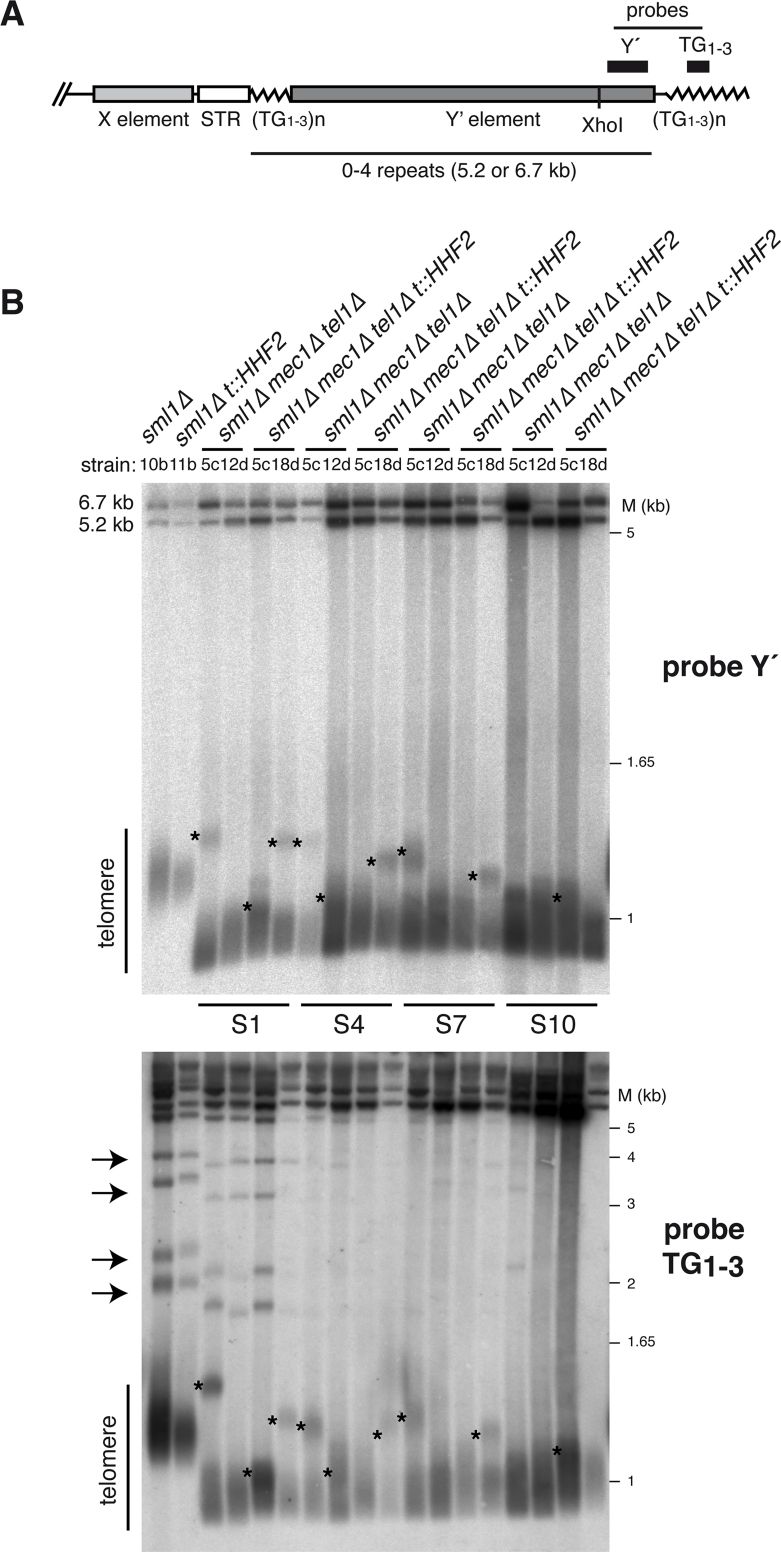
Histone depletion does not affect bulk telomere length in *mec1Δ tel1Δ* cells. **(A)** Schematic structure of yeast telomeres. The position of the Y′ and TG_1-3_ probes used in (B) is shown. **(B)** Telomere length of the strains analyzed in Fig 1E (S1, pre-senescent cells; S4, senescent cells; S7 and S10, surviving cells) as determined by Southern blot of the DNA samples used for T-TFs with either a telomere-proximal Y′ (top gel) or a TG_1-3_ (bottom gel) probe. Subpopulations of long telomeres and X-only telomeres are indicated with asterisks and arrows, respectively.

**Fig 3:**
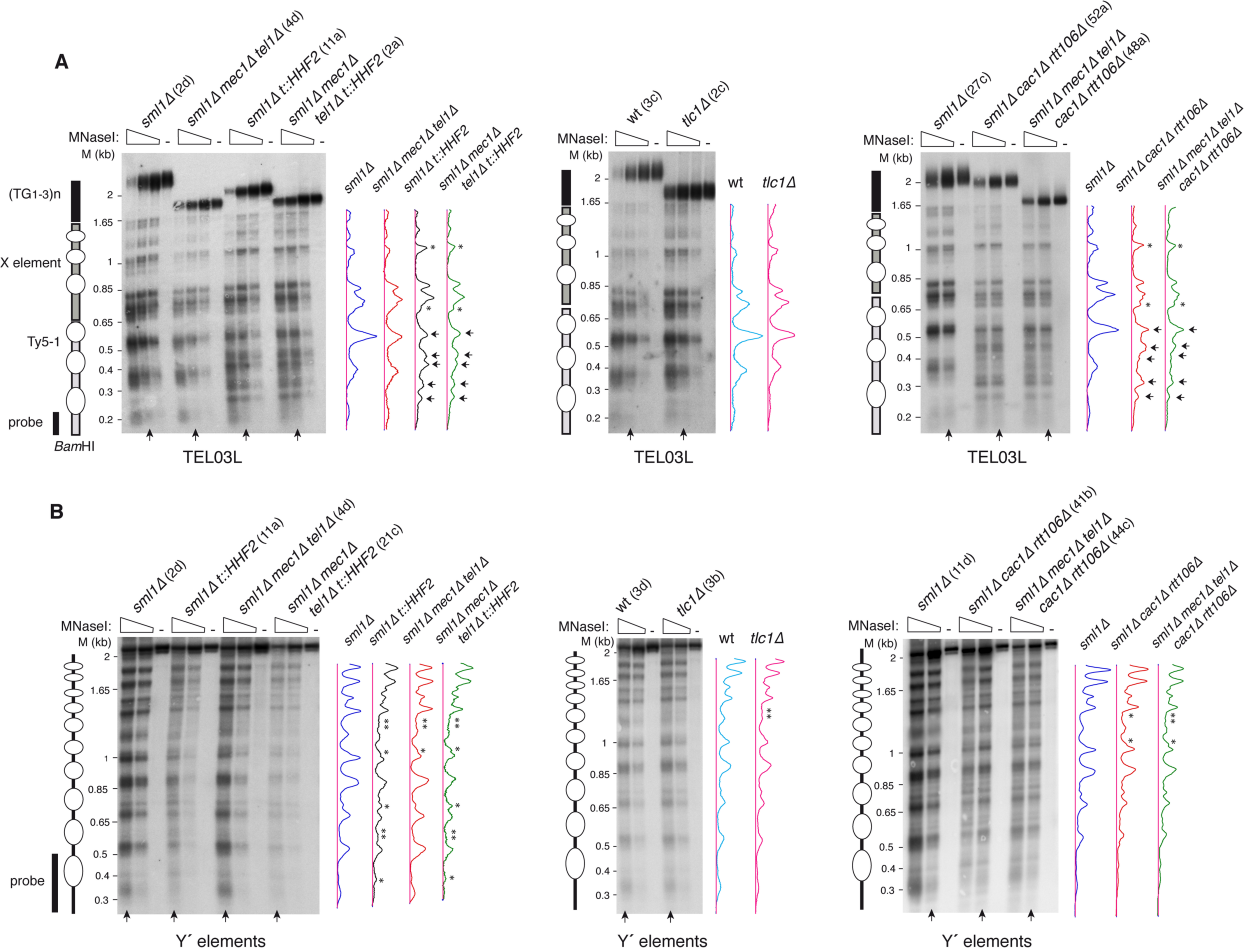
Chromatin analyses of subtelomeric elements in *mec1Δ tel1Δ* and *tlc1Δ* pre-senescent cells. Nucleosome positioning at the left telomere of chromosome III (TEL03L) **(A)** and global nucleosome pattern at the long subtelomeric Y′ elements **(B)** from streak 2-derived cultures of the indicated strains. The profiles of MNaseI accessibility of the indicated lanes (vertical arrow), which display similar MNase digestion, are shown on the right. Subtle (asterisks) and major (arrows) changes in chromatin structure are marked in the profiles. Schemes with the position of nucleosomes from the *Bam*HI site at the Ty5-1 element to the end of TEL03L (A), and the bulk nucleosome pattern of the ~2.3 Kb *Cla*I-*Cla*I fragment from the long Y′ elements (B) are shown on the left. Note that the analysis in (A) shows nucleosome positioning from a specific site at a single telomere, whereas the analysis in (B) shows the pattern of nucleosomes at and around the probe from all long Y′ elements. Ovals indicate the putative nucleosomes inferred from the MNaseI digestion analysis.

T-TFs in *mec1Δ tel1Δ* cells can be suppressed if telomeres are artificially elongated by expressing a Cdc13-Est2 fusion protein [9]. This might explain the disappearance of T-TFs in post-senescent cells (Fig 1E), in which survival is associated with telomere lengthening [7]. Thus, we asked whether histone depletion increases telomere length in *mec1Δ tel1Δ* cells. For a direct comparison, telomere length was analyzed with the same DNA samples used for T-TF analyses. Total DNA was digested with *Xho*I, run into a gel and hybridized to a Y′-specific probe. This assay generates a broad band (~1.25 kb in wild-type cells) encompassing the telomere fragments from Y′-containing telomeres, and two upper bands (~6.7 and ~5.2 kb) that represent the two sizes of tandemly arranged Y′ subtelomeric repeats (Fig 2A) [7,43,44]. Telomere length in *t*::*HHF2* cells was not apparently affected (Fig 2B, top, and S3A Fig), although the analysis of individual chromosomes showed shorter telomeres than those in wild-type cells in some cases (e.g., Fig 3A). As reported, *tel1Δ* but not *mec1Δ* cells displayed short telomeres, whereas *mec1Δ tel1Δ* cells rapidly shortened their telomeres; the *mec1Δ tel1Δ* mutant displayed short telomeres as early as ~25–30 generations after dissection of heterozygous diploids (S3B Fig) [7], which showed wild-type telomeres (S3B Fig) and did not accumulate T-TFs (S2A Fig). Telomere length analysis during pre-senescence, senescence and post-senescence showed a subpopulation of cells in some clones with longer Y′-containing telomeres, which were reduced and disappeared as cells entered into senescence; we do not have an explanation for these events, although they do not seem to be related to the accumulation of T-TFs as they appeared both in *mec1Δ tel1Δ* and *mec1Δ tel1Δ t*::*HHF2* cells (see asterisks in Fig 2B, and S3A and S3B Fig). Indeed, this subpopulation was also detected in *tel1Δ* (S3B Fig). Importantly, histone depletion did not affect bulk telomere length in *mec1Δ tel1Δ t*::*HHF2* cells as compared to *mec1Δ tel1Δ* cells, which were similar from S1 to S10 (Fig 2B, top gel, and S3A and S3B Fig). A similar result was obtained using a TG_1–3_ telomere-specific probe, which also detected telomeres that only contain X subtelomeric elements (Fig 2A and 2B, bottom gel, and S3C Fig). These results suggest that telomere protection against fusions by histone depletion in *mec1Δ tel1Δ t*::*HHF2* cells is not due to bulk telomere elongation. The telomere-specific probe also showed that the amount of X-only telomeres is reduced in *mec1Δ tel1Δ* cells (see arrows in Fig 2B, bottom gel, and S3C Fig). This is a characteristic of type I survivors, which extend telomeres by Y′-element acquisition through Rad51-dependent HR mechanisms [45], although Rad51-independent, Rad59-dependent Y′-acquisition events can also be detected during pre-senescence in cells lacking telomerase [44]. Accordingly, surviving *mec1Δ tel1Δ* cells amplified the Y′ elements (5.2 and 6.7 kb bands), whereas telomere length remained as short as in S1 (Fig 2B, top gel) [7]. The acquisition of Y′ subtelomeric elements might explain the reduction in T-TFs observed in *mec1Δ tel1Δ* survivors (Fig 1E) because the T-TF assay is based on a X-only telomere.

### Histone depletion alters telomeric chromatin in *mec1Δ tel1Δ t*::*HHF2* but not in *tlc1Δ* pre-senescent cells

Next, we wondered whether the accumulation of T-TFs in *mec1Δ tel1Δ* cells, and its suppression by histone depletion are associated with specific changes in the structure of the subtelomeric chromatin. To address this, we analyzed nucleosome positioning at the subtelomeric X element of the left telomere of chromosome III (TEL03L) by indirect–end labeling of MNaseI–treated cells from streak 2–derived cultures (Fig 3A). We also analyzed the chromatin structure of the subtelomeric Y′ elements because X elements are characterized by low nucleosome density [46]. For this, and due to the lack of specific DNA sequences to follow nucleosome positioning at a single Y′ element, global chromatin accessibility was followed with a probe that hybridizes with the long Y′ subtelomeric repeats (Fig 3B). Signal profiles from lanes displaying similar MNase digestion were plotted to compare the patterns of MNaseI accessibility. The absence of Mec1 and Tel1 did not lead to major changes in the structure of either the subtelomeric elements or the Ty5-1 proximal transposon (Fig 3A and 3B; compare *mec1Δ tel1Δ* with the wild type). Remarkably, chromatin structure was also basically unaltered in *tlc1Δ* cells as compared to the wild type (Fig 3A and 3B; only a subtle modification in the Y′ element that was shared by all mutants, and therefore was not associated with T-TFs), despite having reduced histone levels [30]. In line with this result, histone loss seems to be specific for Rap1-targeted promoters in *tlc1Δ* cells [30], suggesting that chromatin assembly is properly regulated under conditions of programmed histone depletion during pre-senescence.

In contrast to *mec1Δ tel1Δ* and *tlc1Δ* cells, histone depletion caused major changes in the chromatin structure of the Ty5-1 transposon (gain or loss of DNA accessibility sites; marked with arrows) and subtle changes in the Y′ and X elements (high background signal and small changes in DNA accessibility sites; marked with asterisks) in both *t*::*HHF2* and *mec1Δ tel1Δ t*::*HHF2* cells (Fig 3A and 3B; compare *t*::*HHF2* and *mec1Δ tel1Δ t*::*HHF2* with the wild type). Finally, we analyzed the same regions in *cac1Δ rtt106Δ* and *mec1Δ tel1Δ cac1Δ rtt106Δ* cells. These mutants displayed similar chromatin changes at the Ty5-1 transposon as *t*::*HHF2* and *mec1Δ tel1Δ t*::*HHF2* cells (Fig 3A; compare the changes marked with arrows in *cac1Δ rtt106Δ* and *mec1Δ tel1Δ cac1Δ rtt106Δ* with those in *t*::*HHF2* and *mec1Δ tel1Δ t*::*HHF2*), as expected for mutants affected in replication-coupled chromatin assembly. However, subtelomeric Y′ chromatin was much less affected in *cac1Δ rtt106Δ* cells than in *t*::*HHF2* cells (Fig 3B; compare the Y′ element MNaseI profiles in *t*::*HHF2* and *mec1Δ tel1Δ t*::*HHF2* with those in *cac1Δ rtt106Δ* and *mec1Δ tel1Δ cac1Δ rtt106Δ*). Therefore, subtelomeric chromatin changes seem to occur specifically in response to induced histone depletion.

### Histone depletion and *mec1Δ tel1Δ* increase NHEJ

**Fig 4:**
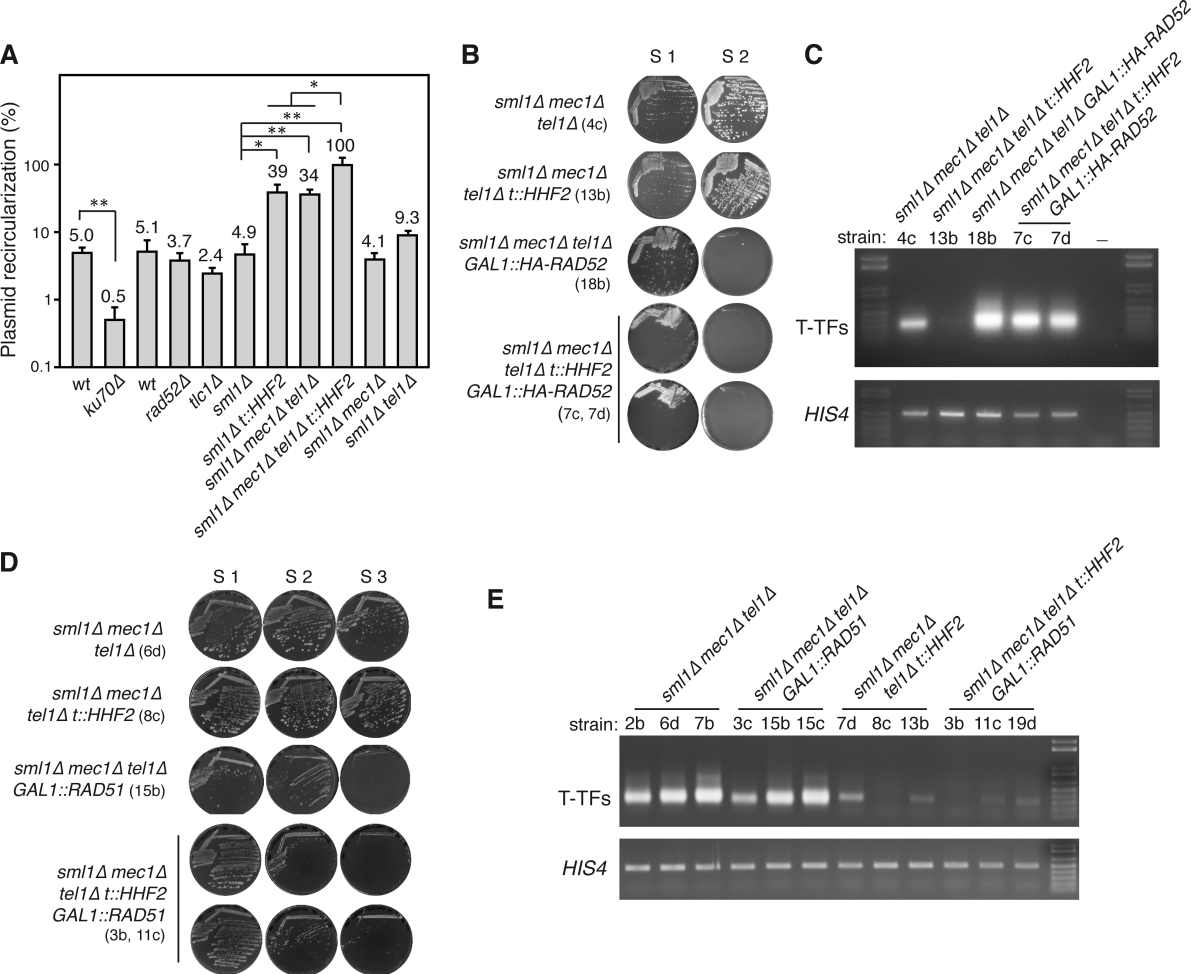
Histone depletion prevents T-TFs in *mec1Δ tel1Δ t*::*HHF2* by stimulating Rad51-independent homologous recombination. **(A)** NHEJ efficiency of the indicated strains as determined by *in vivo* recircularization of a plasmid linearized with a restriction enzyme at a DNA region without homology in the yeast genome. The number of transformants obtained with the linear plasmid was normalized to that obtained by transforming with the same amount of circular plasmid. The average and SEM of 3–4 independent experiments are shown, which include three independent S2 pre-senescent strains. *ku70Δ* and its isogenic wild type were included as a negative control. One (*P*-values <0.05) and two (*P*-values *<*0.005) asterisks indicate statistically significant difference according to an unpaired Student’s *t*-test **(B)** Cell growth analysis of *mec1Δ tel1Δ*, *mec1Δ tel1Δ t*::*HHF2*, *mec1Δ tel1Δ GAL1*::*HA-RAD52*, and *mec1Δ tel1Δ t*::*HHF2 GAL1*::*HA-RAD52* cells (*sml1Δ* background). Strains are indicated in parenthesis. **(C)** T-TF accumulation of the indicated strains from streak 1 biomass as determined by semiquantitative PCR. **(D)** Cell growth analysis of *mec1Δ tel1Δ*, *mec1Δ tel1Δ t*::*HHF2*, *mec1Δ tel1Δ GAL1*::*RAD51*, and *mec1Δ tel1Δ t*::*HHF2 GAL1*::*RAD51* (*sml1Δ* background). Strains are indicated in parenthesis. **(E)** T-TF accumulation of the indicated strains from streak 1 biomass as determined by semiquantitative PCR.

T-TFs in *mec1Δ tel1Δ* cells are NHEJ events [9]. To explore the possibility that histone depletion impairs NHEJ, we used an *in vivo* plasmid-recircularization assay in which the repair of an induced DSB can only occur by NHEJ [47]. For this, S2 pre-senescent cells were transformed with a plasmid linearized at a region with no homology in the yeast genome. Accordingly, cells lacking the NHEJ protein Ku70, but not the recombination protein Rad52 were defective in plasmid recircularization (Fig 4A). Interestingly, whereas the absence of telomerase activity in *tlc1Δ* cells did not affect NHEJ efficiency as compared to wild-type cells, the lack of Mec1 and Tel1 –but not of Mec1 or Tel1 –increased the efficiency of NHEJ repair ~7-fold (Fig 4A), which might explain the high levels of T-TFs in *mec1Δ tel1Δ* cells. Remarkably, histone depletion also increased NHEJ efficiency in *t*::*HHF2* (~9-fold), suggesting that not only the absence of Mec1 and Tel1 but also histone depletion inhibit DNA resection, thus increasing NHEJ frequency. The triple mutant *mec1Δ tel1Δ t*::*HHF2* led to an additive increase (~20-fold) (Fig 4A), suggesting that *mec1Δ tel1Δ* and histone-depleted cells affect DNA resection by different mechanisms. Importantly, the fact that *mec1Δ tel1Δ t*::*HHF2* cells, which have protected telomeres, displayed even higher NHEJ levels than *mec1Δ tel1Δ* cells rules out the possibility that histone depletion prevents T-TFs in *mec1Δ tel1Δ t*::*HHF2* cells by impairing NHEJ.

### Protection against *mec1Δ tel1Δ*-induced telomere fusions by histone depletion requires homologous recombination

A putative protection mechanism against T-TFs might be HR, which competes with NHEJ for DSB processing [48]. In yeast, HR is required to delay senescence early after telomerase inactivation, likely through template switching mechanisms that seem to facilitate the restart of stalled replication forks with the sister chromatid [43–45,49–55]. Thus, a regulated activity of HR at eroded telomeres might be a protective mechanism against telomere fusions. To address this, we first tested the effect of HR on the accumulation of T-TFs in *mec1Δ tel1Δ* by deleting Rad52, which is essential for all types of recombination events in yeast [56]. We detected a significant increase (~5-fold) in T-TFs in *mec1Δ tel1Δ rad52Δ* cells as compared to *mec1Δ tel1Δ* cells (Fig 1D and S4A Fig). We then dissected diploids heterozygous for *mec1Δ*, *tel1Δ*, *hhf1Δ*, *hhf2Δ* and *rad52Δ*, but we failed to obtain *mec1Δ tel1Δ t*::*HHF2 rad52Δ* cells, likely due to a combination of mutations affecting genome integrity. To overcome this problem, diploids containing *HA-RAD52* under control of the *GAL1* promoter were dissected in galactose-containing plates and streaked in glucose-containing plates. Although this tagged form of Rad52 was hardly functional (S4B Fig), it allowed us to obtain some *mec1Δ tel1Δ t*::*HHF2 GAL1*::*HA-RAD52* spores that, similar to *mec1Δ tel1Δ GAL1*::*HA-RAD52* cells, senesced in the first streak (Fig 4B), in line with HR playing a major role in the replication of telomeres during pre-senescence [55]. This residual activity of HA-Rad52 might explain why the frequency of T-TFs was lower in *mec1Δ tel1Δ GAL1*::*HA-RAD52* cells than in *mec1Δ tel1Δ rad52Δ* cells (Fig 1D). Importantly, the amount of T-TFs in *mec1Δ tel1Δ* cells was not reduced by inducing histone depletion in the absence of Rad52 activity (Figs 1D and 4C), indicating that suppression of T-TFs in *mec1Δ tel1Δ t*::*HHF2* cells completely depends on HR. Again, T-TF accumulation in the absence of Rad52 was not associated with changes in bulk telomere length, which was similar in S1 pre-senescent *mec1Δ tel1Δ* and *mec1Δ tel1Δ t*::*HHF2* cells regardless of the presence or absence of Rad52 (S4C Fig). Therefore, HR is necessary during pre-senescence not only to help stalled replication forks but also to prevent T-TFs by a mechanism that further requires a reduction in the pool of available histones.

In order to gain a deeper insight into the mechanism of HR that protects telomeres from fusions in *mec1Δ tel1Δ t*::*HHF2* cells, we analyzed the role of the strand exchange protein Rad51. As for *rad52Δ* strains, we failed to obtain *mec1Δ tel1Δ t*::*HHF2 rad51Δ* spores and thus decided to dissect diploids heterozygous for *RAD51/ GAL1*::*RAD51*; in this case, some *mec1Δ tel1Δ t*::*HHF2 GAL1*::*RAD51* spores germinated even in glucose-containing medium. The absence of Rad51 accelerated the entry into senescence of *mec1Δ tel1Δ* cells (Fig 4D) [53,57], although the effect was less pronounced than the one observed in the absence of Rad52 (compare Fig 4B and 4D), as previously reported for telomerase defective cells [53]. In addition, the absence of Rad51 prevented the appearance of *mec1Δ tel1Δ* survivors after streak S2 (Fig 4D), as expected for type I survivors [45]. Importantly, the absence of Rad51 did not increase the levels of T-TFs in *mec1Δ tel1Δ t*::*HHF2* cells (Figs 1D and 4E), indicating that Rad51 is dispensable for protecting telomeres by histone depletion.

### HR-dependent and -independent mechanisms prevent T-TFs in telomerase-deficient cells

Our results suggest that the inability of *mec1Δ tel1Δ* cells to induce histone depletion during pre-senescence leads to the formation of telomere fusions. This raises the possibility that the Mec1-mediated histone depletion that occurs in cells lacking telomerase during pre-senescence prevents T-TFs. Indeed, the absence of Mec1 increases the frequency of T-TFs in *tlc1Δ* cells [9]. To investigate this possibility, *tlc1Δ* cells were transformed with a multicopy plasmid expressing the four core histones. However, this genetic strategy hardly increased the levels of histones and did not lead to T-TFs in *tlc1Δ* background (S5 Fig). This is not unexpected, considering the number of mechanisms that prevent histone overexpression [58].

**Fig 5:**
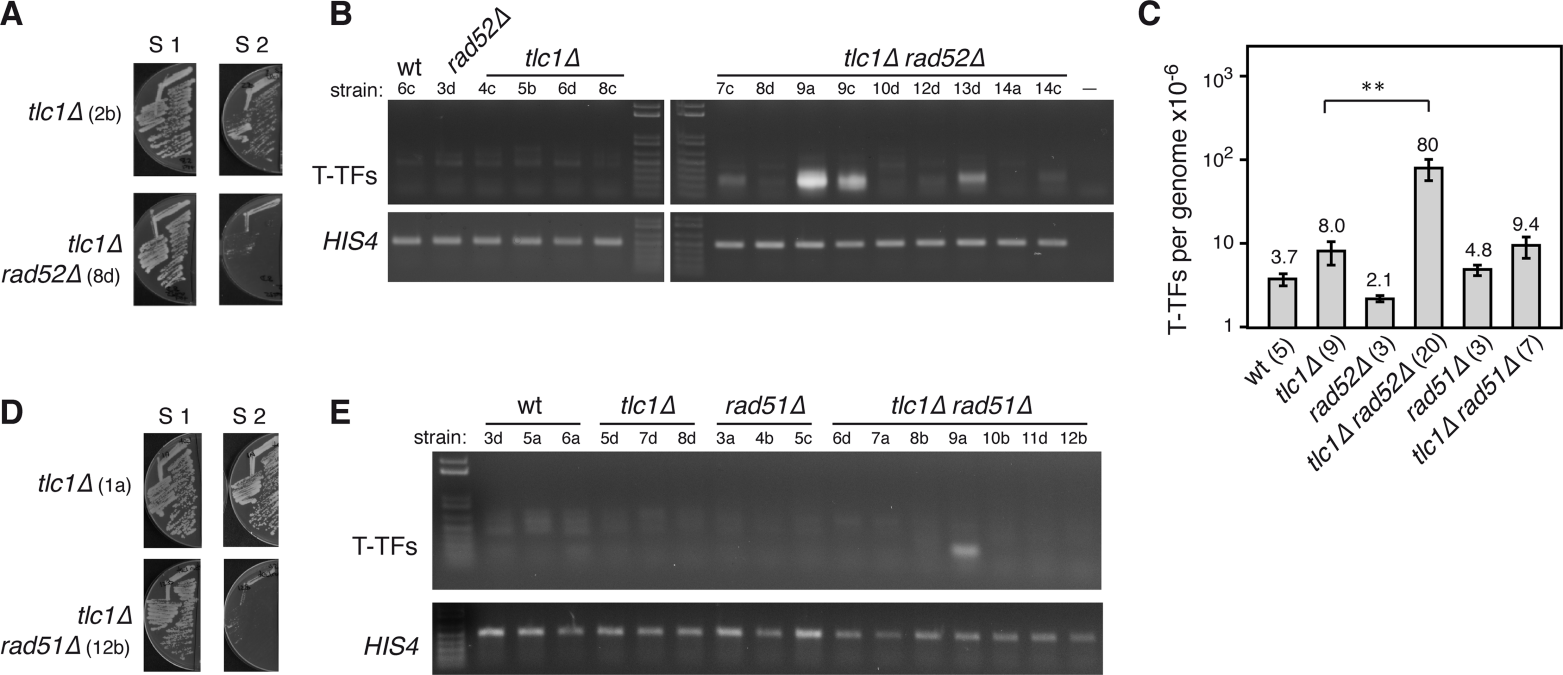
Rad52 partially prevents T-TFs in *tlc1Δ* cells. **(A, D)** Cell growth comparative analyses of *tlc1Δ* with *tlc1Δ rad52Δ* (A) and *tlc1Δ rad51Δ* (D) cells. Strains are indicated in parenthesis. Haploid strains obtained from diploids heterozygous for those markers were dissected on plates containing rich medium and restreaked on the same medium. **(B, E)** Effect of *rad52Δ* (B) and *rad51Δ* (E) on the accumulation of T-TFs in *tlc1Δ* cells as determined by semiquantitative PCR from streak 1 biomass of the indicated strains (shown below each genotype) **(C)** T-TF frequency of the indicated strains as determined by quantitative PCR from S1 streaks. The average and SEM are shown. The number of DNA samples from independent strains, which include those used for semiquantitative PCR and telomere length analyses, is shown in parenthesis for each genotype. Two asterisks indicate a statistically significant difference according to a Student’s *t*-test (Mann-Whitney) (*P*-values *<*0.001).

Since the mechanism by which histone depletion prevents T-TFs in *mec1Δ tel1Δ t*::*HHF2* cells depends on HR, we asked if Rad52 is required to prevent T-TFs in *tlc1Δ* cells. The absence of Rad52 shortened dramatically the pre-senescent state of *tlc1Δ* (Fig 5A) [57], as observed for *mec1Δ tel1Δ* cells (Fig 4B). We thus analyzed T-TFs from the streak S1 biomass and found that the absence of Rad52 in *tlc1Δ rad52Δ* cells increased ~10-fold the frequency of T-TFs as compared to *tlc1Δ* cells (Fig 5B and 5C). This indicates that HR also prevents T-TFs in the absence of telomerase activity. However, this increase in T-TFs was variable and in most cases small as compared to that observed by the lack of Rad52 activity in *mec1Δ tel1Δ t*::*HHF2 GAL1*::*HA-RAD52* cells (Fig 1D; compare Figs 4C and 5B), suggesting that additional HR-independent mechanisms that protect the telomeres in *tlc1Δ* cells are lost in *mec1Δ tel1Δ* cells. To further compare the protective role of HR in *tlc1Δ* and *mec1Δ tel1Δ* cells, we analyzed the effects of *rad51Δ*. As reported, Rad51 was also essential to maintain the pre-senescence state of cells lacking telomerase (Fig 5D) [57]. Moreover, most *tlc1Δ rad51Δ* clones did no accumulate T-TFs (Fig 5C and 5E), except for 2 out of 16 strains in which the absence of Rad51 led to an accumulation of telomere fusions (2 × 10^−4^/genome). Indeed, we cannot discard that these events also occurred in a low number of *mec1Δ tel1Δ t*::*HHF2* clones lacking Rad51 activity, as only three clones could be analyzed (Fig 4E). To address whether the variability in T-TFs was associated with telomere length, DNA samples from the streak S1 biomass were used to analyze T-TFs and telomere length (S6A and S6B Fig). In general, telomeres were slightly longer in *tlc1Δ rad52Δ* and *tlc1Δ rad51Δ* cells than in *tlc1Δ* cells. However, there was no apparent correlation between bulk telomere length and T-TFs in *tlc1Δ* cells lacking HR activity.

## Discussion

One of the means by which ATR/Mec1 and ATM/Tel1 preserve genome integrity is preventing T-TFs. Despite the importance of understanding how genetic instability accumulates in the absence of these tumor suppressor genes, the mechanisms by which they carry out this protective role remain unknown. We show that T-TFs in *mec1Δ tel1Δ* cells can be suppressed by inducing a partial reduction in the pool of available histones. This suppression, together with the fact that Mec1 is required for histone depletion in pre-senescent *tlc1Δ* cells [30], which have protected telomeres [9], suggest that T-TFs accumulate in *mec1Δ tel1Δ* cells due in part to their inability to induce histone depletion. We show that the absence of Mec1 and Tel1 strongly augments DSB repair by NHEJ, which might also contribute to the high frequency of T-TFs in *mec1Δ tel1Δ* cells. However, histone depletion does not prevent telomere fusions by inhibiting NHEJ. Rather, histone depletion prevents telomere fusions by facilitating the recombinational processing of unprotected telomeres through a Rad51-independent mechanism. This recombination mechanism is different from the main mechanisms that facilitate the elongation of critically short telomeres during pre-senescence or that amplify Y′ subtelomeric elements in surviving cells, which do not require reduced levels of histones and are highly dependent on Rad51.

Mec1 and Tel1 regulate DNA resection [59]. Using a plasmid recircularization assay we observed that the absence of Mec1 and Tel1 –but not of Mec1 or Tel1 –strongly increases DSB repair by NHEJ, suggesting that they have essential and redundant functions in DNA resection that are revealed only after eliminating both factors. According to this result, T-TF accumulation in *mec1Δ tel1Δ* cells might result as a consequence of defective DNA resection at telomeres, which would shift the balance between NHEJ and HR toward NHEJ. In addition, this result raises the possibility that histone depletion prevents telomere fusions by inhibiting NHEJ. However, histone depletion also increases NHEJ, not only in *t*::*HHF2* (~8-fold) cells but also in *mec1Δ tel1Δ t*::*HHF2* cells (~20-fold), making it unlikely that histone depletion prevents T-TFs by directly inhibiting NHEJ. Instead, our results show that histone depletion prevents T-TFs in pre-senescent *mec1Δ tel1Δ t*::*HHF2* cells by a Rad51-independent mechanism of HR. This is in apparent contradiction with the observed increase in NHEJ in histone-depleted cells. However, histone-depleted cells are proficient in HR [31,39], suggesting that histone depletion facilitates HR under conditions of impaired DNA resection.

Histone depletion in yeast impairs the stability of advancing replication forks, leading to fork breakage and rescue by a Rad51-independent HR mechanism [39]. This phenotype is shared with the replication coupled–chromatin assembly *asf1Δ* and *cac1Δ rtt106Δ* mutants [37,40,60]. Therefore, histone depletion in *mec1Δ tel1Δ t*::*HHF2* might increase the amount of recombinogenic lesions at telomeres, leading to a local high concentration of recombination factors that would compete with the NHEJ machinery thus preventing T-TFs. We have discarded this possibility by showing that *cac1Δ rtt106Δ* did not prevent the accumulation of T-TFs in *mec1Δ tel1Δ* background. Further, this possibility assumes that HR is limiting at telomeres in the absence of Mec1 and Tel1, which seems not to be the case for two reasons: first, HR is proficient in maintaining the pre-senescence state in *mec1Δ tel1Δ* cells (Fig 4B); and second, post-senescence *mec1Δ tel1Δ* surviving cells require HR (Fig 4B and 4D) [9]. Therefore, HR is efficient at telomeres in the absence of Mec1 and Tel1, and histone depletion hardly affects this efficiency, as *mec1Δ tel1Δ* and *mec1Δ tel1Δ t*::*HHF2* cells display similar profiles of senescence entry and survivor formation. We observed only a slight increase in the doubling time of pre-senescent cells that might be associated with the suppression of telomere fusions.

**Fig 6:**
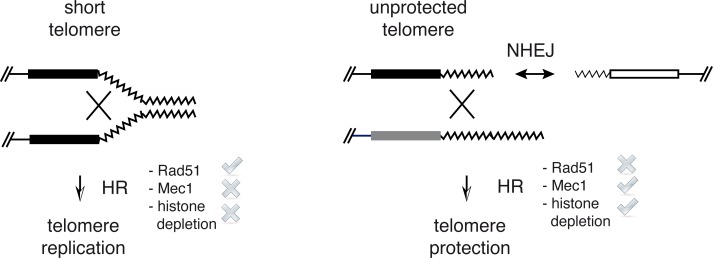
Model for telomere protection by induced histone depletion in *mec1Δ tel1Δ* cells. Rad51-dependent HR is required to delay senescence early after telomerase inactivation, likely through template switching mechanisms that seem to facilitate the restart of stalled replication forks with the sister chromatid (left). Cells lacking Mec1 and Tel1 maintain this delay despite they do not induce histone depletion. However, they accumulate unprotected telomeres that are prone to fuse with each other. These fusions might be stimulated by defects in DNA resection. Induced histone depletion in *mec1Δ tel1Δ* cells facilitates the processing of unprotected telomeres by a Rad51-independent mechanism–likely BIR–thus preventing NHEJ and T-TFs (right). Cells lacking telomerase also prevent telomere fusions through a Rad51-independent HR mechanism, opening the possibility that the Mec1-dependent histone reduction that accompanies senescence is required for telomere protection.

HR maintains the proliferative pre-senescent state of cells lacking telomerase by facilitating the recombinational restart of stalled replication forks at telomeres [55]. This is achieved through different recombination mechanisms. The accumulation of the non-coding RNA TERRA as R-loops at short telomeres has been shown to promote the HR-dependent restart of stalled replication forks [61]. Replication fork restart at telomeres can occur both by break-induced replication (BIR), which deals with one-ended DSBs [36,44,54], and sister-chromatid recombination (SCR) [62]. These mechanisms are highly dependent on Rad51, and accordingly the lack of Rad51 accelerates the entry into senescence of cells lacking telomerase [57]. Rad51-independent, Rad59-dependent BIR events can also be detected, but its relevance in maintaining the proliferative pre-senescent state of cells lacking telomerase is reduced in comparison with Rad51-dependent events as inferred from the slight effect on senescence entry induced by the absence of Rad59 [44]. The fact that HR is able to maintain the proliferative pre-senescent state but not to prevent T-TFs in *mec1Δ tel1Δ* cells suggests that the recombination events that protect telomeres from fusions differ from those that allow telomere DNA replication before senescence. We think that HR acts upon different substrates in these two processes: stalled replication forks, to help replication at telomeres during pre-senescence, and DSBs, to compete with NHEJ and prevent T-TFs (Fig 6). They differ in that the latter further requires Mec1 and histone depletion and can operate in the absence of Rad51. Although less efficiently, BIR can operate in the absence of Rad51 [63]. Therefore, we suggest that histone depletion promotes the processing of unprotected telomeres by BIR. Actually, although telomere protection was not associated with changes in bulk telomere length, we cannot discard that fusions affect a subpopulation of critically short telomeres, and that HR promotes their protection by BIR-induced lengthening.

How histone depletion facilitates the recombinational processing of unprotected telomeres is currently unknown. One possibility is that the more accessible chromatin structure of the unprotected telomeres in *mec1Δ tel1Δ t*::*HHF2* cells favors its processing by HR, thus reducing the potential substrate for NHEJ and consequently, the frequency of T-TFs. A more accessible chromatin might facilitate recombination-limiting steps. In line with this, the HR proteins Rad51 and Rad52, but not the NHEJ protein Ku80, bind with higher efficiency to DSBs under conditions of reduced levels of histones in yeast [64], and histone depletion by knock down of human SLBP shifts the balance between NHEJ and HR during DSB repair toward HR [65]. In addition, chromatin disruption by either histone depletion or mutations in Spt6 and Spt4 stimulates Rad51-independent HR by BIR [39,66]. Finally, defective chromatin assembly in *mec1Δ tel1Δ cac1Δ rtt106Δ* cells hardly affects the subtelomeric chromatin as compared to histone depletion in *mec1Δ tel1Δ t*::*HHF2* cells, and it does not protect telomeres. However, programmed histone depletion in pre-senescent cells is not associated with changes in the subtelomeric chromatin, arguing against the idea that histone depletion facilitates the recombinational processing of telomeres by making subtelomeric chromatin more accessible to the recombination machinery. Indeed, most T-TFs in *mec1Δ tel1Δ* involve the joining of the telomere repetitive tracts [9]. Alternatively, histone depletion might affect telomere anchoring to the nuclear envelope and/or the folding back of the telomere as a first step to facilitate the access of the recombination machinery, as both processes have been shown to repress recombination at telomeres [67,68]. Further studies will be required to elucidate how histone depletion promotes the recombinational processing of unprotected telomeres, which should include the genes regulated by Rap1 relocalization and histone depletion in response to telomere shortening in *tlc1Δ* cells during pre-senescence [30]. Nevertheless, it must be stressed that the fact that induced histone depletion prevents T-TFs in *mec1Δ tel1Δ* cells does not necessarily mean that this is the mechanism that operates in *tlc1Δ* cells, although the observation that Rad51-independent HR is also required for telomere protection against fusions in *tlc1Δ* cells supports this possibility. Furthermore, the lack of Rad52 leads to higher levels of T-TFs in *mec1Δ tel1Δ t*::*HHF2* cells than in *tlc1Δ* cells. Again, this observation might reflect not different protection mechanisms but a higher frequency of telomere breakage in *mec1Δ tel1Δ* than in *tlc1Δ* cells, with the subsequent accumulation of DSBs to be processed by NHEJ. Accordingly, Mec1 is required for replication fork stability [69], specifically in hard-to-replicate sites [70]. Alternatively, this difference could be associated with the accumulation of NHEJ observed in *mec1Δ tel1Δ t*::*HHF2* cells as compared to *tlc1Δ* cells.

In response to telomere shortening human fibroblasts reduce the levels of histones and chromatin assembly factors, which in turn disrupts chromatin integrity to reinforce the activation of the ATM and ATR pathways that accompany the senescence process [29]. Likewise, histone depletion is a hallmark of replicative senescence and aging in yeast [30,71], suggesting that it has been evolutionarily conserved in these processes [72]. Induced histone depletion in yeast impairs chromatin integrity, DNA replication, chromosome segregation and DNA topology, leading to genetic instability and checkpoint activation [38,39,73]. Hence, we suggest that histone depletion has a dual role in genome integrity during pre-senescence in yeast. Histone depletion leads to DNA damage helping to activate senescence. On the other hand, histone depletion prevents the deleterious consequences of telomere fusions and subsequent fusion-bridge-breakage cycles during the temporal window in which telomeres are unprotected.

## Materials and methods

### Yeast strains, plasmids and growth conditions

Yeast strains used to generate the spores analyzed in this study are listed in S1 Table. Deletion mutants were constructed by a PCR-based strategy [74]. All analyses were performed with haploid strains derived from diploids heterozygous for *mec1Δ* and *tel1Δ* and grown at 30°C in 2% glucose-rich medium (YPAD)–unless 2% galactose-rich medium is indicated–and containing 5 μg/ml doxycycline (except for the *tlc1Δ* strain and its isogenic wild-type strain). Plasmid p413TARtetH4 is a centromeric plasmid in which the expression of *HHF2* is under control of the doxycycline-inducible *tet* promoter [31]. pRS426 is a *URA3*-based multicopy plasmid [75]. p314N795 is a *TRP1*-based centromeric plasmid for rat glucocorticoid receptor (rGR) [76]. p426-H342A2B is a multicopy plasmid expressing the four core histones. For its construction the *HHT1-HHF1* and *HTA1-HTB1* genomic loci were PCR amplified, cut with *Xho*I/*Eco*RI or *Eco*RI/*Not*I, respectively, and inserted into the *Xho*I-*Not*I site of pRS426 by a triple ligation. Oligonucleotides for PCR amplification are listed in S2 Table.

### Plasmid-recircularization assay

The repair of an induced DSB by NHEJ was performed as described [47]. Cells were transformed with 600 ng of plasmid p314N795 that had either been linearized at the rGR ORF with *Nco*I or was uncut, and the efficiency of plasmid recircularization was determined as the number of transformants obtained with the linear plasmid relative to that obtained with the uncut plasmid.

### Telomere-telomere fusions analysis

Telomere-telomere fusions (T-TFs) were analyzed by semiquantitative and quantitative PCR analyses as reported [9]. Briefly, ~100 ng of *Sau*3A-treated genomic DNA extracted by standard protocols from asynchronous cultures was PCR-amplified for semiquantitative analyses using a primer from the X element of chromosome XV-L and a primer from the Y′ element of chromosome V-R (coordinates 183–207 and 576759–576783, respectively; Stanford Genome Database). A DNA fragment from *HIS4* was PCR-amplified as input control. T-TFs and *HIS4* were PCR-amplified using 35 or 20 cycles, respectively, under the conditions previously reported. Oligonucleotides for PCR amplification are listed in S2 Table. Quantitative analyses were performed by real-time PCR by using the same amount of *Sau*3A-treated genomic DNA, the oligonucleotides described above for semiquantitative analyses and the PCR conditions described previously [9]. The frequency of T-TFs per genome was calculated with the formula: T-TFs/genome = 2^–N^ / N = Ct (T-TFs)–Ct (*HIS4*). Prior to applying this formula, the curves representing the increasing amounts of DNA for the two products as a function of the number of PCR cycles were confirmed to be parallel (i.e., the slope of the curve representing the log of the input amount versus ΔCt was < 0.1).

### Telomere length analysis

Total DNA from asynchronous cultures was extracted by standard protocols. DNA samples were digested with *Xho*I and run in 1.2% TBE 1× agarose gels for 15 hours at 2 V/cm. Gels were blotted onto Hybond-XL membranes and hybridized either at 65°C with a ^32^P-labeled PCR fragment containing 600 bp from *Xho*I to the centromere-distal end of the subtelomeric Y’ element (probe Y′) or at 37°C with a TG_1–3_ oligo labeled at the 5′ terminus with ATP (γ-^32^P) and T4 polynucleotide kinase (TG_1–3_ probe). Oligonucleotides are listed in S2 Table. All signals were quantified in a Fuji FLA5100 with the ImageGauge analysis program.

### Chromatin analysis by MNaseI digestion

Nucleosome positioning at TEL03L was determined by micrococcal nuclease (MNaseI) digestion and indirect end-labelling [31]. MNaseI–treated DNA was digested with *Bam*HI, resolved in a 1.5% agarose gel, blotted onto a HybondTM-XL membrane and probed with a 230-bp ^32^P-labeled PCR fragment located at 60 bp from the *Bam*HI site that reveals nucleosome positioning from this restriction site to TEL03L. Chromatin structure of the long subtelomeric Y′ elements was determined by the same method, except that MNaseI–treated DNA was digested with *Cla*I, and probed with a 517-bp ^32^P-labeled fragment located at 412 bp from the centromere-proximal *Cla*I site that reveals bulk chromatin accessibility (mono-, di-, tri- … nucleosomes) at and around the probed region. Oligonucleotides for PCR amplification are listed in S2 Table. MNaseI profiles were generated with the ImageGauge analysis program.

### Western blot

Yeast protein extracts were prepared using the TCA protocol as described [31] and run on a 15% sodium dodecyl sulfate-polyacrylamide gel. Histone H4 and Pgk1 were detected with the rabbit polyclonal ab10158 (Abcam) and the mouse polyclonal 22C5D8 (Invitrogen) primary antibodies, respectively, and either fluorophore-conjugate or peroxidase-conjugate secondary antibodies. Total protein was determined by using the TFX Stain-Free FastCast Acrylamide kit (Biorad) [77]. Bands were visualized and quantified using either the Odyssey infrared Imaging System (Licor) or the ChemiDoc MP image system (Biorad).

### Chromatin fractionation

Chromatin fractionation was performed as described for young yeast cells [71] with some modifications. Samples (30 ml) from mid-log phase cultures were collected by centrifugation, washed with cold 0.1mM Tris pH 9.4, 10mM DTT, and incubated for 15 min in 1 ml of the same buffer on ice. Cells were then washed with cold spheroplasting buffer (20mM Hepes pH 7.4, 1.2mM sorbitol, Roche Complete EDTA free protease inhibitor cocktail) and incubated with 1 ml of the same buffer with 210 μg zymoliase 20T for 1 h at 30°C. The spheroplasts were collected, washed twice with cold washing buffer (20mM Tris pH 7.4, 20mM KCl, 1M sorbitol, 0.1μM spermine, 0.25μM spermidine, protease inhibitors), and resuspended in 1 ml lysis buffer (20mM Tris pH 7.4, 20mM KCl, 1M sorbitol, 0.1μM spermine, 0.25μM spermidine, 1% Triton X-100, protease inhibitors) for 5 min on ice. An aliquot (80 μl) was removed for the total sample, and the remaining sample was centrifuged for 15 min at 13000 g at 4°C to separate soluble (supernatant) and chromatin-enriched (pellet) fractions. Each pellet was washed with 0.5 ml cold lysis buffer and resuspended in 80 μl of water, and chromatin, soluble and total samples were mixed with SDS buffer for western blot analyses. Similar volumes were loaded for each sample, and similar cell equivalents of the chromatin and soluble fractions were loaded for the fractionation controls.

### Statistical analyses

Statistical analyses were performed using the Prism software (Graphpad). Numerical data that underlies graphs and statistical analyses are provided in S3 Table.

## Supporting information

S1 Fig Induced histone depletion hardly affects *mec1Δ tel1Δ* cell growth. **(A)** Histone H4 and Pgk1 levels at chromatin and soluble fractions of the indicated strains from S1-derived cultures. The average and range from two independent experiments is shown on the bottom. **(B)** Cell growth analysis of wild type, *t*::*HHF2*, *mec1Δ tel1Δ* and *mec1Δ tel1Δ t*::*HHF2* cells (*sml1Δ* background). Strains are indicated in parenthesis. See text for details. **(C)** Cell viability from S1, S3 and S6-derived cultures of *sml1Δ mec1Δ tel1Δ* (strains 43D, 5D and 53D), *sml1Δ mec1Δ tel1Δ t*::*HHF2* (strains 7D, 8C and 40A) and *tlc1Δ* (strains 3C, 4B and 5A) cells. **(D–F)** Cell growth (D), viability (E), and doubling time (F) of wild type, *t*::*HHF2*, *mec1Δ tel1Δ* and *mec1Δ tel1Δ t*::*HHF2* cells (*sml1Δ* background) from streak 2-derived cultures. Cell growth analysis in (D) was performed by plating ten-fold serial dilutions from the same number of mid-log phase cells. Cell viability was determined as the frequency of cells from an asynchronous liquid culture able to form colonies. The total amount of cells was counted in a Burker chamber. The average and SEM of three independent strains are plotted.
(TIF)

S2 Fig Histone depletion-mediated protection of telomeres in *mec1Δ tel1Δ t*::*HHF2* is independent of Htz1. **(A)** T-TFs in *mec1Δ tel1Δ* and *mec1Δ tel1Δ t*::*HHF2* cells (*sml1Δ* background) from spore-inoculated cultures and diploids heterozygous for the indicated markers. The result from four (spores) and two (diploids) independent strains (indicated below each genotype) is shown. **(B)** T-TFs accumulation in *sml1Δ mec1Δ tel1Δ htz1Δ* cells from streak 1 biomass from the indicated strains. T-TFs from *sml1Δ mec1Δ tel1Δ* and *sml1Δ mec1Δ tel1Δ t*::*HHF2* cells from streak 1 biomass was included as control. **(C, D)** Cell growth analysis of *mec1Δ tel1Δ htz1Δ* cells (C) and *mec1Δ tel1Δ cac1Δ rtt106Δ* cells (D) (*sml1Δ* background) from the indicated strains. Diploids heterozygous for those markers were dissected on rich-medium plates, and cells were streaked for several times on the same medium (S1 to S7).
(TIF)

S3 Fig Telomere length analyses in *mec1Δ tel1Δ* and *mec1Δ tel1Δ t*::*HHF2*. **(A, B)** Telomere length of the indicated strains as determined by southern blot using either a telomere-proximal Y′ probe **(A, B)** or a TG_1-3_ probe **(C)**. See legend to Fig 2 for more details.
(TIF)

S4 Fig Role of HR on telomere fusions and telomere length in *mec1Δ tel1Δ* cells. **(A)** T-TF accumulation in *sml1Δ mec1Δ tel1Δ* and *sml1Δ mec1Δ tel1Δ rad52Δ* strains (indicated below each genotype) from streak S1 biomass, as determined by semi-quantitative PCR. **(B)** HA-Rad52 is not functional. Cell growth was determined for wild-type and *GAL1*::*HA-RAD52* strains in glucose and galactose medium in the absence or presence of MMS at the indicated concentrations. **(C)** T-TF accumulation in *mec1Δ tel1Δ* cells is not associated with changes in bulk telomere length. Telomere length of the indicated strains from streak S1 biomass was determined by probing DNA samples from Fig 4C with a telomere-proximal Y′ probe. All samples were run in the same gel.
(TIF)

S5 Fig Histone overexpression in *tlc1Δ* cells. **(A)** Histone H4 levels in *mec1Δ tel1Δ* and *tlc1Δ* cells, and in *tlc1Δ* cells transformed with either p426-H3.4.2A.2B (histone overexpression) or pRS426 (empty vector) from streak 1-derived cultures as determined by western blot. The amount of histone H4 was normalized to the amount of Pgk1. The average and range of 2 independent strains are shown, as well as the image of one the blots. **(B)** T-TFs in *tlc1Δ* cells transformed with either p426-H3.4.2A.2B (histone overexpression) or pRS426 (empty vector) from streak 1-derived cultures. Similar results were obtained with 8 more spores. *tlc1Δ* strains were obtained from *TLC1/tlc1Δ* diploids transformed with the corresponding plasmid.
(TIF)

S6 Fig T-TF variability *in tlc1Δ rad52Δ* cells is not associated with differences in bulk telomere length. **(A, B)** T-TF accumulation **(A)** and telomere length **(B)** of the indicated strains from S1 biomass, as determined by semiquantitative PCR and southern blot (using a Y′-specific probe), respectively. Total DNA was split into two samples for T-TF and telomere length analyses. Asterisks in (B) indicate subpopulations of long telomeres.
(TIF)

S1 Table *Saccharomyces cerevisiae* strains used in this study. (DOCX)

S2 Table Oligonucleotides used in this study. (DOCX)

S3 Table Numerical data underlying graphs. (XLSX)
