# Supplementary figures and images for: Histone depletion prevents telomere fusions in pre-senescent cells
Source: PLoS Genet. 2018 Jun 7;14(6):e1007407. doi: 10.1371/journal.pgen.1007407 (PMC5991667; doi:10.1371/journal.pgen.1007407)

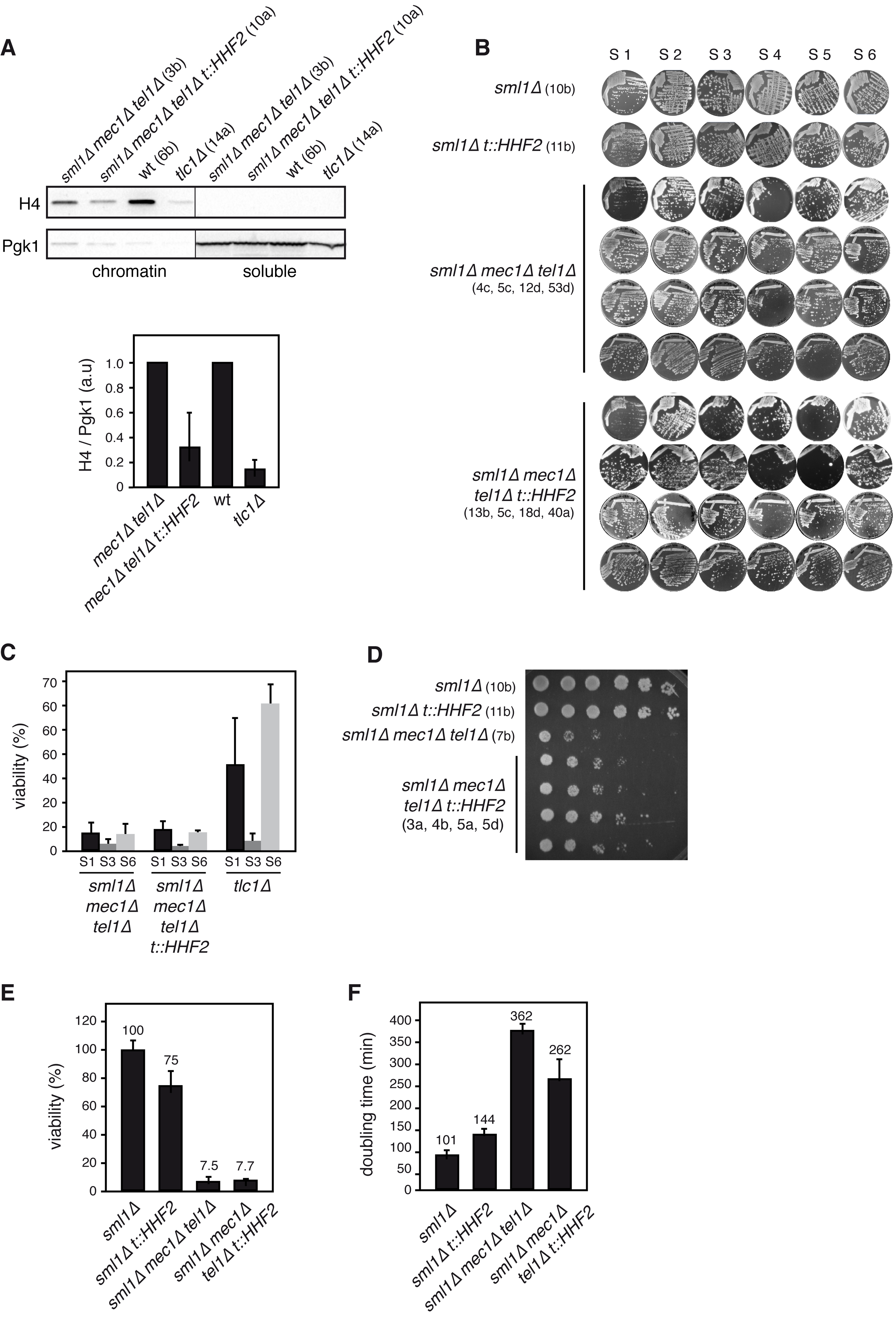

Supplement: S1 Fig — (A) Histone H4 and Pgk1 levels at chromatin and soluble fractions of the indicated strains from S1-derived cultures. The average and range from two independent experiments is shown on the bottom. (B) Cell growth analysis of wild type, t::HHF2, mec1Δ tel1Δ and mec1Δ tel1Δ t::HHF2 cells (sml1Δ background). Strains are indicated in parenthesis. See text for details. (C) Cell viability from S1, S3 and S6-derived cultures of sml1Δ mec1Δ tel1Δ (strains 43D, 5D and 53D), sml1Δ mec1Δ tel1Δ t::HHF2 (strains 7D, 8C and 40A) and tlc1Δ (strains 3C, 4B and 5A) cells. (D–F) Cell growth (D), viability (E), and doubling time (F) of wild type, t::HHF2, mec1Δ tel1Δ and mec1Δ tel1Δ t::HHF2 cells (sml1Δ background) from streak 2-derived cultures. Cell growth analysis in (D) was performed by plating ten-fold serial dilutions from the same number of mid-log phase cells. Cell viability was determined as the frequency of cells from an asynchronous liquid culture able to form colonies. The total amount of cells was counted in a Burker chamber. The average and SEM of three independent strains are plotted. (TIF) [file pgen.1007407.s001.tif]

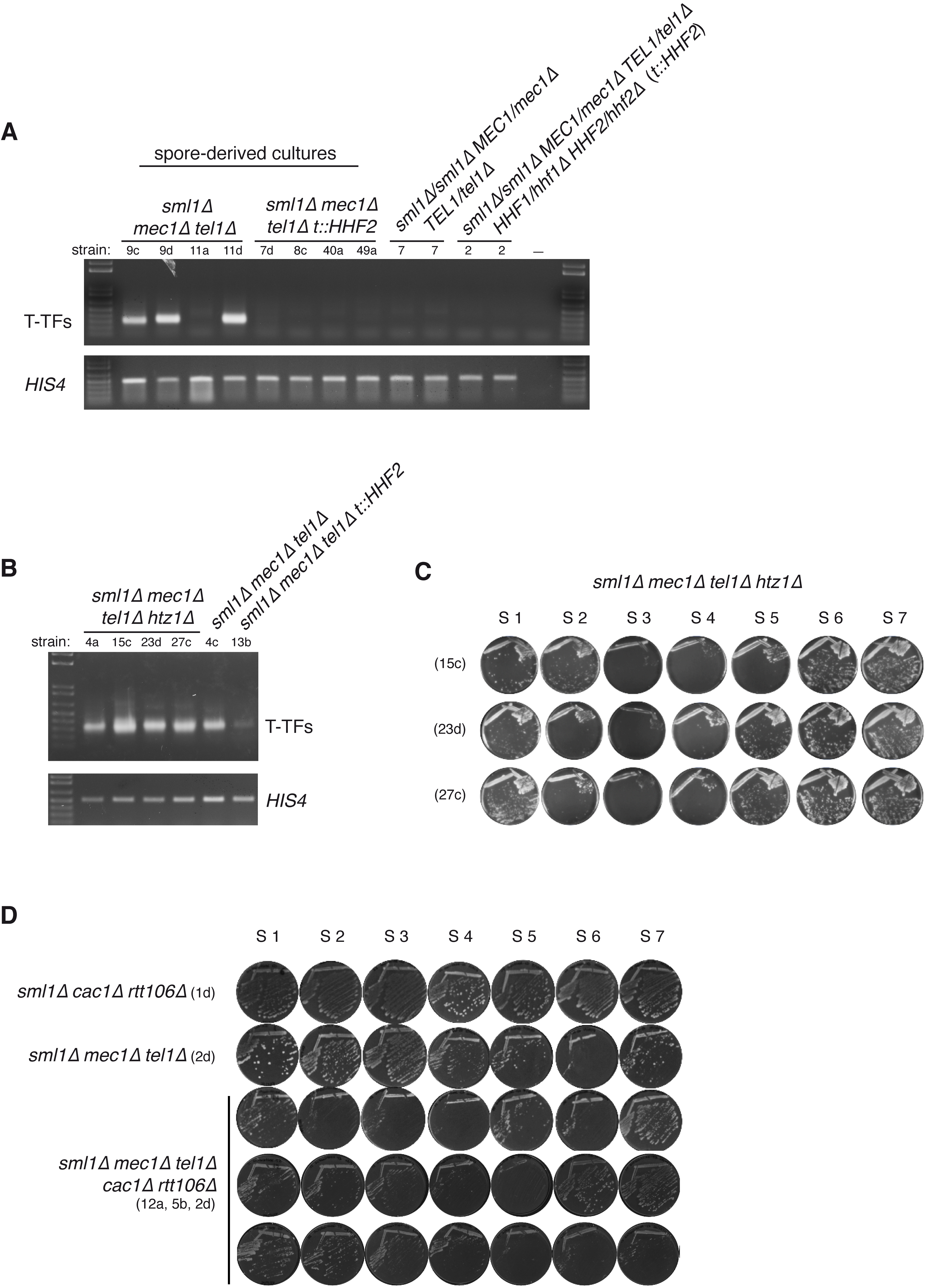

Supplement: S2 Fig — (A) T-TFs in mec1Δ tel1Δ and mec1Δ tel1Δ t::HHF2 cells (sml1Δ background) from spore-inoculated cultures and diploids heterozygous for the indicated markers. The result from four (spores) and two (diploids) independent strains (indicated below each genotype) is shown. (B) T-TFs accumulation in sml1Δ mec1Δ tel1Δ htz1Δ cells from streak 1 biomass from the indicated strains. T-TFs from sml1Δ mec1Δ tel1Δ and sml1Δ mec1Δ tel1Δ t::HHF2 cells from streak 1 biomass was included as control. (C, D) Cell growth analysis of mec1Δ tel1Δ htz1Δ cells (C) and mec1Δ tel1Δ cac1Δ rtt106Δ cells (D) (sml1Δ background) from the indicated strains. Diploids heterozygous for those markers were dissected on rich-medium plates, and cells were streaked for several times on the same medium (S1 to S7). (TIF) [file pgen.1007407.s002.tif]

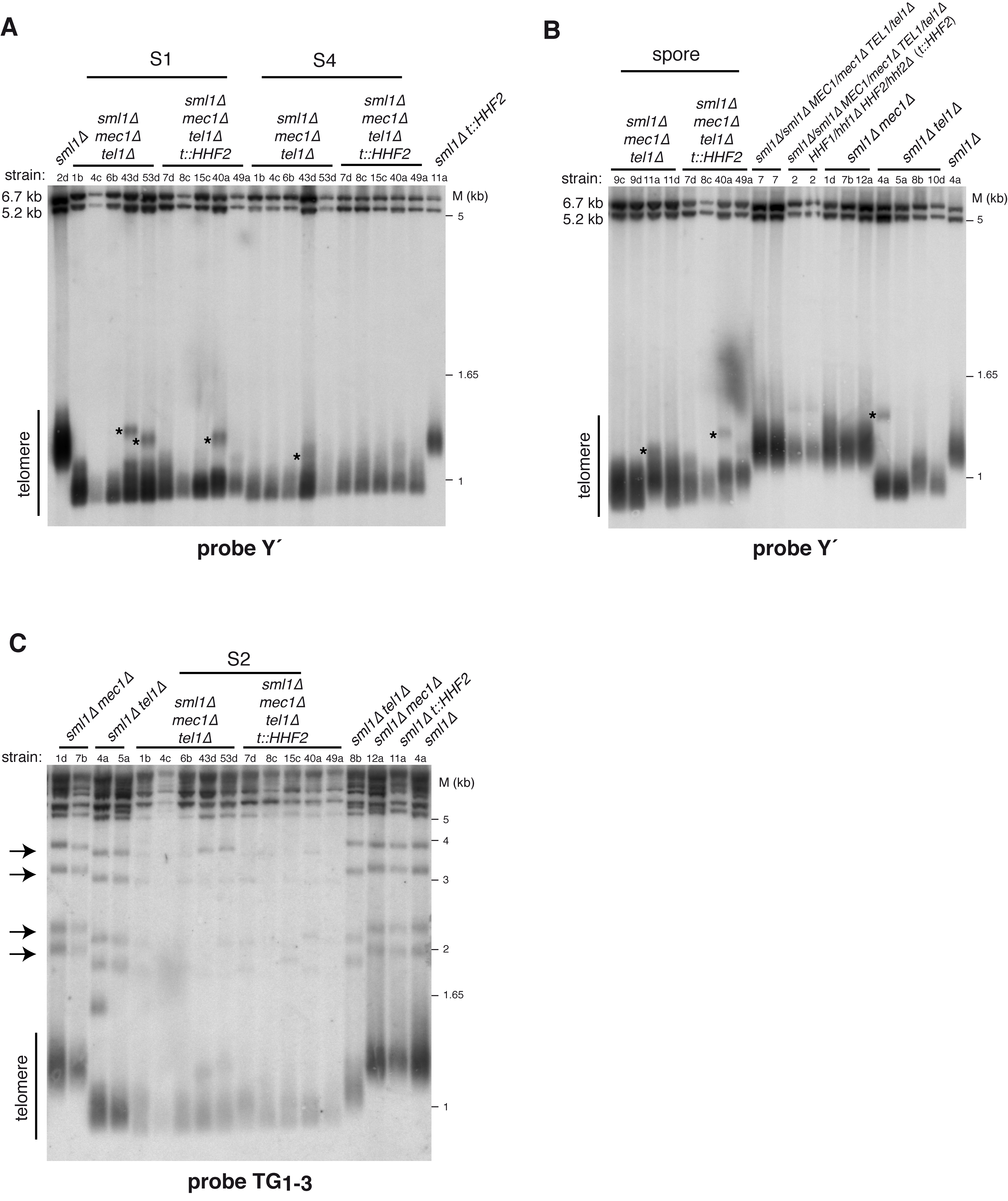

Supplement: S3 Fig — (A, B) Telomere length of the indicated strains as determined by southern blot using either a telomere-proximal Y′ probe (A, B) or a TG1-3 probe (C). See legend to Fig 2 for more details. (TIF) [file pgen.1007407.s003.tif]

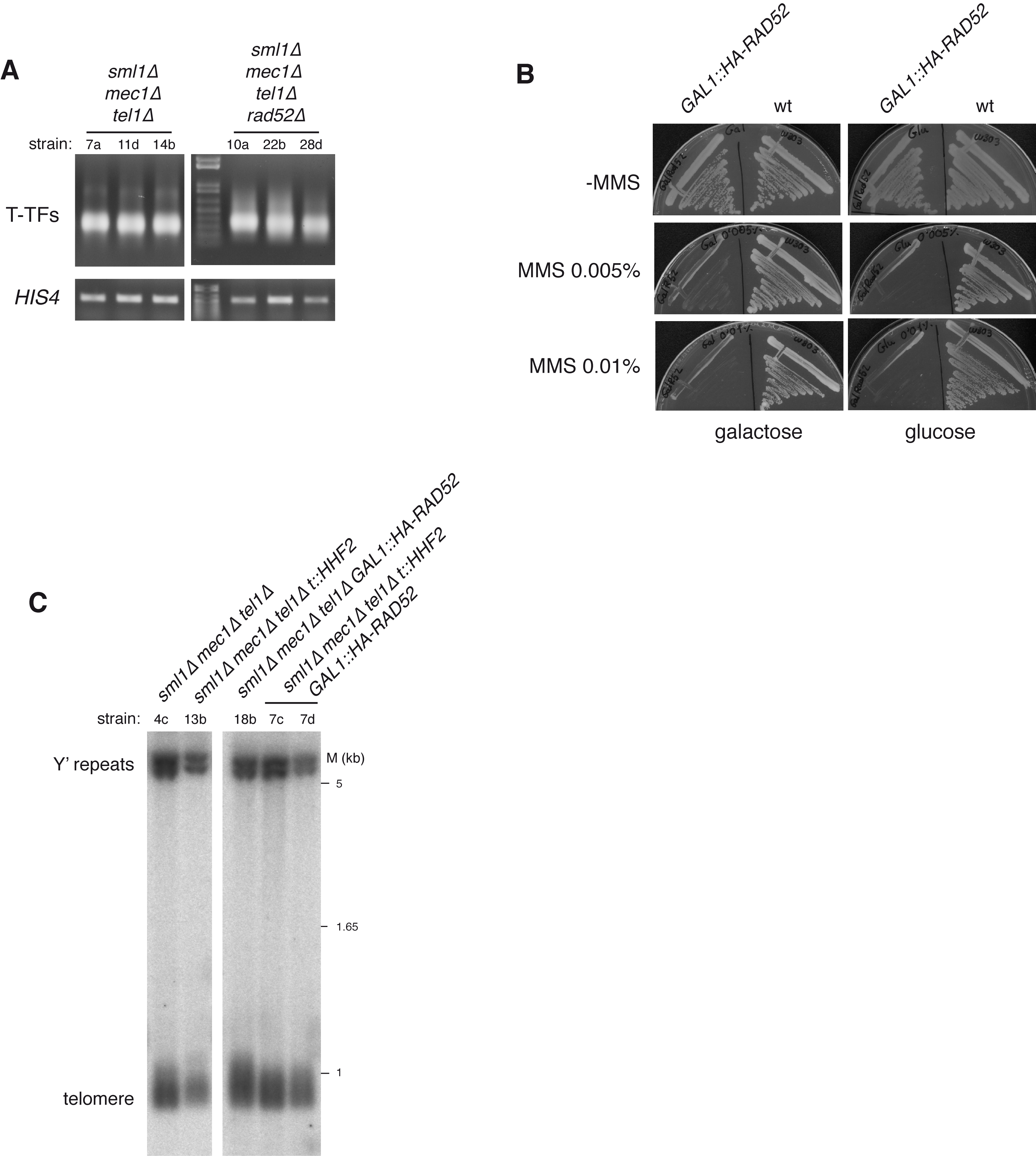

Supplement: S4 Fig — (A) T-TF accumulation in sml1Δ mec1Δ tel1Δ and sml1Δ mec1Δ tel1Δ rad52Δ strains (indicated below each genotype) from streak S1 biomass, as determined by semi-quantitative PCR. (B) HA-Rad52 is not functional. Cell growth was determined for wild-type and GAL1::HA-RAD52 strains in glucose and galactose medium in the absence or presence of MMS at the indicated concentrations. (C) T-TF accumulation in mec1Δ tel1Δ cells is not associated with changes in bulk telomere length. Telomere length of the indicated strains from streak S1 biomass was determined by probing DNA samples from Fig 4C with a telomere-proximal Y′ probe. All samples were run in the same gel. (TIF) [file pgen.1007407.s004.tif]

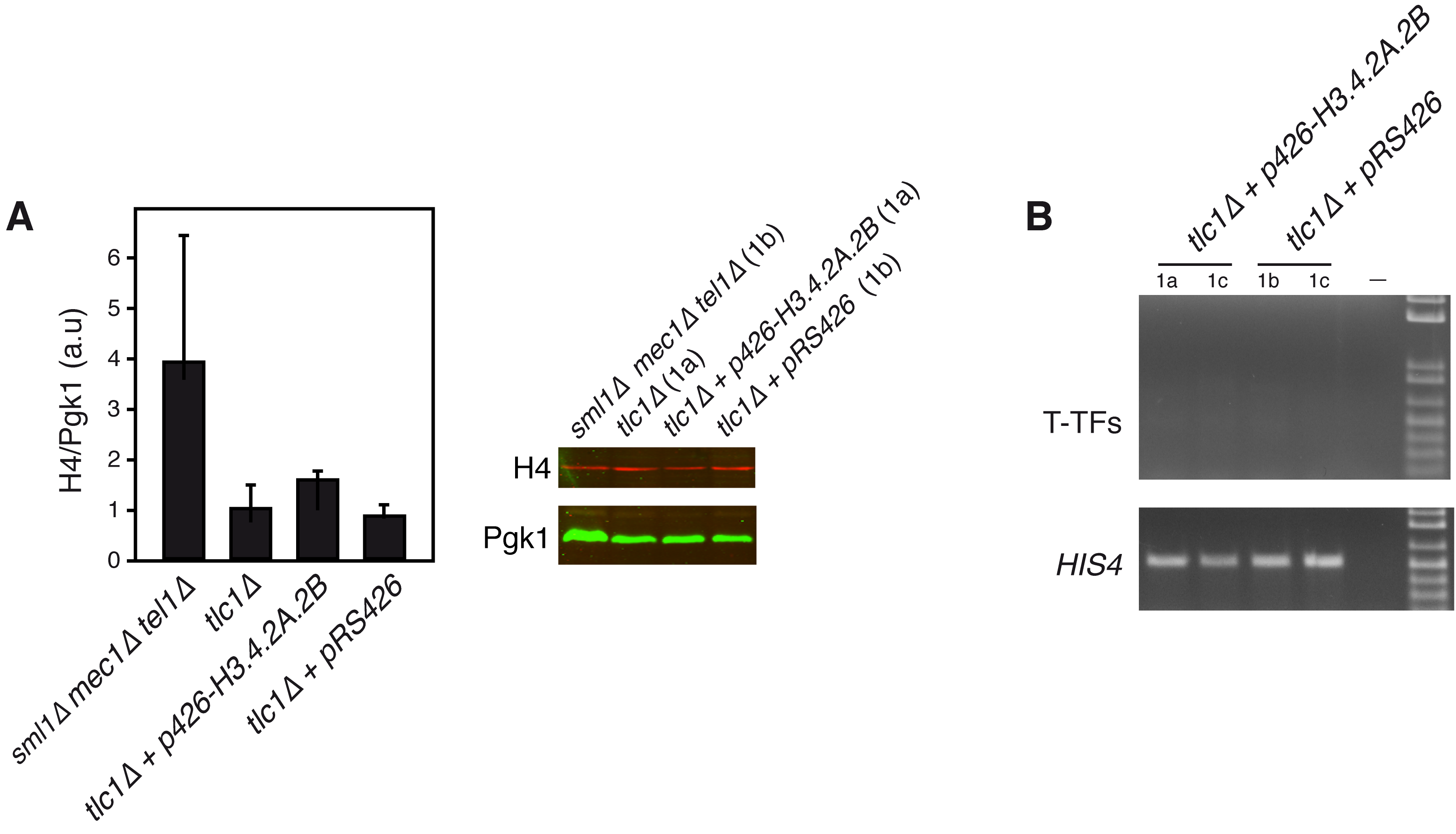

Supplement: S5 Fig — (A) Histone H4 levels in mec1Δ tel1Δ and tlc1Δ cells, and in tlc1Δ cells transformed with either p426-H3.4.2A.2B (histone overexpression) or pRS426 (empty vector) from streak 1-derived cultures as determined by western blot. The amount of histone H4 was normalized to the amount of Pgk1. The average and range of 2 independent strains are shown, as well as the image of one the blots. (B) T-TFs in tlc1Δ cells transformed with either p426-H3.4.2A.2B (histone overexpression) or pRS426 (empty vector) from streak 1-derived cultures. Similar results were obtained with 8 more spores. tlc1Δ strains were obtained from TLC1/tlc1Δ diploids transformed with the corresponding plasmid. (TIF) [file pgen.1007407.s005.tif]

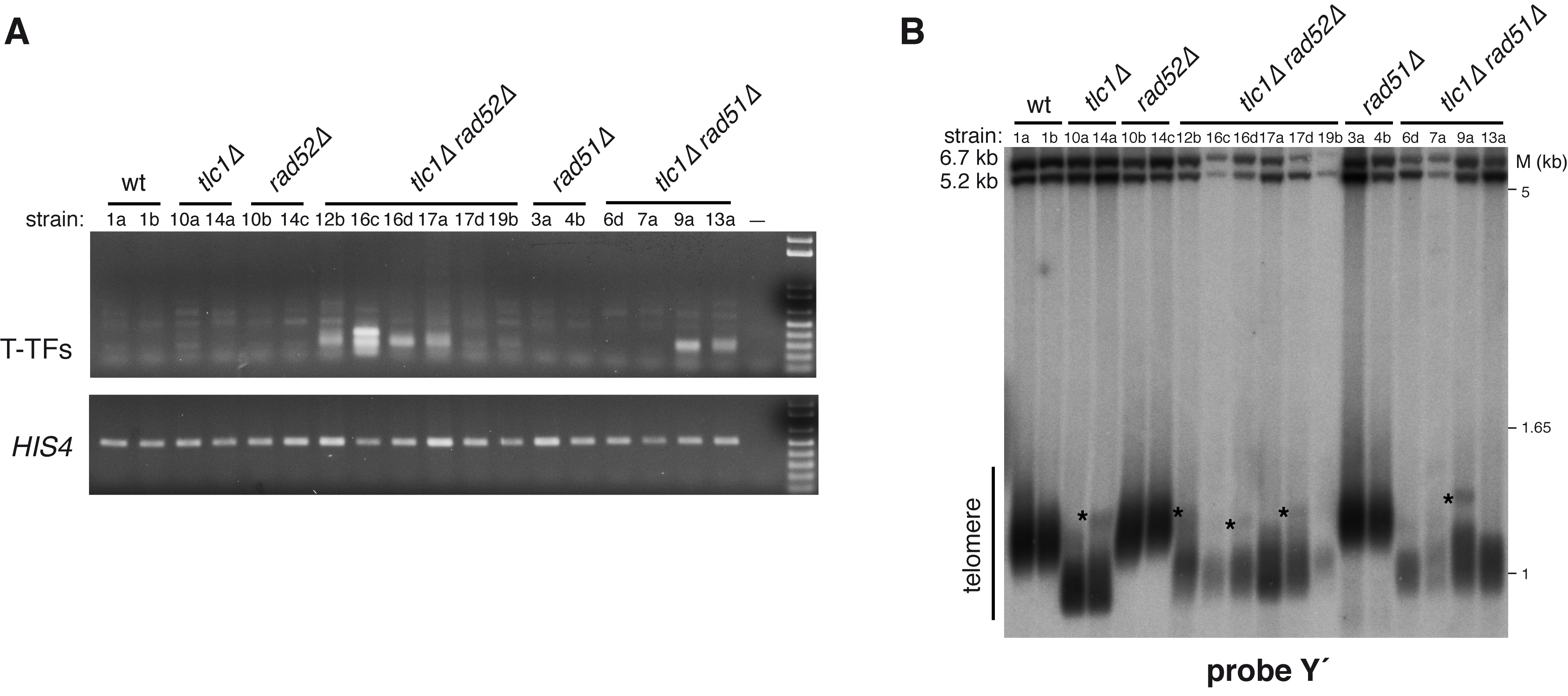

Supplement: S6 Fig — (A, B) T-TF accumulation (A) and telomere length (B) of the indicated strains from S1 biomass, as determined by semiquantitative PCR and southern blot (using a Y′-specific probe), respectively. Total DNA was split into two samples for T-TF and telomere length analyses. Asterisks in (B) indicate subpopulations of long telomeres. (TIF) [file pgen.1007407.s006.tif]
