# Supplementary material for: Histone depletion prevents telomere fusions in pre-senescent cells
Source: PLoS Genet. 2018 Jun 7;14(6):e1007407. doi: 10.1371/journal.pgen.1007407 (PMC5991667; doi:10.1371/journal.pgen.1007407)
Supplement: S1 Table — (DOCX) [file pgen.1007407.s007.docx]

**S1 Table. *Saccharomyces cerevisiae* strains used in this study**

| **Diploids** | | | | | |
| --- | --- | --- | --- | --- | --- |
| **Strain** | **Cross** | **Genotype** | **Ref.** | **Dissected spores** | **Figure** |
| D1 | H8 x H15 | *MATa/α sml1∆::URA3/sml1∆::HIS3 MEC1/mec1∆::TRP1 TEL1/tel1∆::HygMX4 SET1/set1∆::NatMX4* | This work | *mec1∆tel1∆ (1b,4d)* | 1C,D; 3A,B; S5A |
| D2 | H8x H16 | *MATa/α HHF1/hhf1∆::HygMX4 HHF2/hhf2∆::KanMX4 (p413TARtetH4) sml1∆::URA3/sml1∆::URA3 MEC1/mec1∆::LEU2 TEL1/tel1∆::HygMX4* | This work | *mec1∆tel1∆ (5c,12d,43d,53d)*  *mec1∆tel1∆t::HHF2 (2a,3a,18d,24a,15c,40a,49a)* | 1A,C-E; 2B; 3A; 4A; S1B,C; S2A; S3A-C |
| D3 | H14 x H19 | *MATa/α HHF1/hhf1∆::HygMX4 HHF2/hhf2∆::KanMX4 (p413TARtetH4) sml1∆::URA3/sml1∆::URA3 MEC1/mec1∆::LEU2 TEL1/tel1∆::NatMX4* | This work | *mec1∆tel1∆t::HHF2 (11c)* | 1B |
| D4 | H16 x H20 | *MATa/α HHF1/hhf1∆::HygMX4 HHF2/hhf2∆::KanMX4 (p413TARtetH4) sml1∆::URA3/sml1∆::URA3 MEC1/mec1∆::LEU2 TEL1/tel1∆::HygMX4 ade2-1/ade2-1::ADE2 RAD52/rad52::KanMX4::GAL1-3HA-RAD52* | This work | *mec1∆tel1∆ (4c)*  *mec1∆tel1∆t::HHF2 (10a,13b)*  *mec1∆tel1∆GAL1::3HA-RAD52 (18b)*  *mec1∆tel1∆t::HHF2GAL1::3HA-RAD52 (7c,7d)* | 1D; 4B,C; S1A,B; S2A; S4C |
| D5 | H12 x H20 | *sml1∆::URA3/sml1∆::URA3 MEC1/mec1∆::LEU2 TEL1/tel1∆::HygMX4 ade2-1/ade2-1::ADE2 RAD52/rad52::KanMX4::GAL1-3HA-RAD52* |  | *mec1∆tel1∆GAL1::3HA-RAD52 (1a,1b,7d)* | 1D |
| D6 | H19 x H21 | *MATa/α RAD5/rad5-535 HHF1/hhf1∆::HygMX4 HHF2/hhf2∆::KanMX4 (p413TARtetH4) sml1∆::URA3/sml1∆::URA3 MEC1/mec1∆::LEU2 TEL1/tel1∆::NatMX4 MAD2/mad2∆::KanMX4* | This work | *mec1∆tel1∆ (2a)*  *mec1∆tel1∆t::HHF2 (5c)* | 1D,E; 2B; S1B |
| D7 | H9 x H14 | *MATa/α sml1∆::URA3/sml1∆::URA3 MEC1/mec1∆::LEU2 TEL1/tel1∆::HygMX4* | This work | *mec1∆tel1∆ (1b,2b,3b,3d,4c,4d,5d,6b,7b,9c,9d,11a,11d)* | 1A,B; 4A; S1A,C-F; S2A; S3A-C |
| D8 | H9 x H17 | *MATa/α HHF1/hhf1∆::HygMX4 HHF2/hhf2∆::KanMX4 (p413TARtetH4) sml1∆::URA3/sml1∆::URA3 MEC1/mec1∆::LEU2 TEL1/tel1∆::HygMX4* | This work | *mec1∆tel1∆t::HHF2 (21c)* | 3B |
| D9 | H17 x H18 | *MATa/α hhf1∆::HygMX4/hhf1∆::HygMX4 hhf2∆::KanMX4/hhf2∆::KanMX4 (p413TARtetH4) SML1/sml1∆::URA3 MEC1/mec1∆::LEU2 TEL1/tel1∆::NatMX4* | This work | *mec1∆tel1∆t::HHF2 (3a,4b,5a,5d)* | S1D-F |
| D10 | H22 x H24 | *MATa/α TLC1/tlc1∆::HygMX4 RAD52/rad52∆::HygMX4* | This work | *w303 (6c,8a)*  *tlc1∆ (4c,5b,6d,8c)*  *rad52∆ (3d,4b,4d)*  *tlc1∆rad52∆ (7c,8d,9a,9c,10d,12d,13d,14a,14c)* | 1D; 5A,B |
| D11 | H23 x H24 | *MATa/α TLC1/tlc1∆::HygMX4 RAD52/rad52∆::HygMX4* | This work | *w303 (1a,1b,6b)*  *tlc1∆ (10a,14a,21a)*  *rad52∆ (10b,14c)*  *tlc1∆rad52∆ (1c,1d,12b,12c,15c,16c,16d,17a,17d,19b,19d)* | 1B,D; 5C; S1A; S6A,B |
| D12 | H22 x H25 | *MATa/α TLC1/tlc1∆::HygMX4 RAD51/rad51∆::kanMX4 BAR1/bar1∆* | This work | *w303 (3d,5a,6a)*  *tlc1∆ (5d,7d,8d)*  *rad51∆ (3a,4b,5c)*  *tlc1∆rad51∆ (6d,7a,8b,9a,10b,11d,12b,13a,14d)* | 1D; 5C-E; S6A,B |
| D13 | H26x H31 | *MATa/α TLC1/tlc1∆::HygMX4* | This work | *w303 (3c,3d)*  *tlc1∆ (2c,3b)* | 3A,B |
| D14 | H27 x H31 | *MATa/α TLC1/tlc1∆::HygMX4* | This work | *w303 (1a,1c,2b)*  *tlc1∆ (3c,4b,5a)* | 4A; S1C |
| D15 | H27 x H31 | *MATa/α TLC1/tlc1∆::HygMX4 (pRS426 or p426-H3.4.2A.2B)* | This work | *tlc1∆ (1a,1d,12c)*  *tlc1∆+pRS426 (1b,1c)*  *tlc1∆+p426-H3.4.2A.2B (1a,1c)* | 1A; S5A,B |
| D16 | H8 x H28 | *MATa/α sml1∆::URA3/sml1∆::URA3 MEC1/mec1∆::LEU2 TEL1/tel1∆::HygMX4 HTZ1/htz1∆::HygMX4* | This work | *mec1∆tel1∆htz1∆ (4a,15c,23d,27c)* | 1D; S2B,C |
| D17 | H29 x H30 | *MATa/α sml1∆::URA3/sml1∆::HIS3 MEC1/mec1∆::TRP1 TEL1/tel1∆::HygMX4 SET1/set1∆::NatMX4 RAD52/rad52∆::HygMX4* | This work | *mec1∆tel1∆ (6d,7a, 10d,11d,12b,14b)*  *mec1∆tel1∆rad52∆ (10a,22b,28d,29b,38c,42c)* | 1D; S4A |
| D18 | H8 x H32 | *MATa/α sml1∆::URA3/sml1∆::URA3 MEC1/mec1∆::LEU2 TEL1/tel1∆::HygMX4 CAC1/cac1∆::KanMX4 RTT106/rtt106∆::HygMX4* | This work | *sml11∆ (11d,27c)*  *cac1∆rtt106∆ (2a,8d,41b,52a)*  *mec1∆tel1∆ (2d,9a,21c)*  *mec1∆tel1∆ cac1∆rtt106∆ (8a,14a,16d,44c,48a)* | 1D,F; 3A,B; S2D |
| D19 | H8 x H33 | *MATa/α sml1∆::URA3/sml1∆::URA3 MEC1/mec1∆::LEU2 TEL1/tel1∆::HygMX4 CAC1/cac1∆::KanMX4 RTT106/rtt106∆::HygMX4* | This work | *cac1∆rtt106∆ (1d,6a,14a)*  *mec1∆tel1∆ (6d,14c)*  *mec1∆tel1∆ cac1∆rtt106∆ (2d,5b,12a,14d,20c)* | 1D,F; S2D |
| D20 | H19 x H37 | *MATa/α HHF1/hhf1∆::HygMX4 HHF2/hhf2∆::KanMX4 (p413TARtetH4) SML1/sml1∆::URA3 MEC1/mec1∆::LEU2 TEL1/tel1∆::NatMX4 RAD51/TRP1::GAL1-RAD51* | This work | *mec1∆tel1∆t::HHF2 (7d,8c)*  *mec1∆tel1∆t::HHF2GAL1-RAD51 (3b,11c,19d)* | 1A,D; 4A,D,E; S1C; S2A; S3A-C |
| D21 | H8 x H36 | *MATa/α SML1/sml1∆::URA3 MEC1/mec1∆::LEU2 TEL1/tel1∆::NatMX4 RAD51/TRP1::GAL1-RAD51* | This work | *mec1∆tel1∆ (2b,6d,7b,11b)*  *mec1∆tel1∆GAL1-RAD51 (3c,15b,15c)* | 1D; 4D,E |
| D22 | H9 x H37 | *MATa/α SML1/sml1∆::URA3 MEC1/mec1∆::LEU2 TEL1/tel1∆::NatMX4 RAD51/TRP1::GAL1-RAD51* | This work | *mec1∆tel1∆GAL1-RAD51 (16c)* | 1D |

| **Haploids** | | | |
| --- | --- | --- | --- |
| **Code** | **Strain** | **Genotype** | **Ref.** |
| H1 | w303s1-2D | *MATa sml1∆::URA3* | This work |
| H2 | w303s1-3C | *MATa sml1∆::URA3* | This work |
| H3 | w303s1-4A | *MATα sml1∆::URA3* | This work |
| H4 | w303sml1-10B | *MATa sml1∆::URA3* | [1] |
| H5 | wtH4s1-11B | *MATa hhf1∆::HygMX4 hhf2∆::KanMX4 (p413TARtetH4) sml1∆::URA3* | [2] |
| H6 | wtH4s1-11A | *MATa hhf1∆::HygMX4 hhf2∆::KanMX4 (p413TARtetH4) sml1∆::URA3* | This work |
| H7 | wtH4s1-16D | *MATa hhf1∆::HygMX4 hhf2∆::KanMX4 (p413TARtetH4) sml1∆::URA3* | This work |
| H8 | ws1t1-4A | *MATa sml1∆::URA3 tel1∆::HygMX4* | This work |
| H9 | ws1t1-5A | *MATα sml1∆::URA3 tel1∆::HygMX4* | This work |
| H10 | ws1t1-8B | *MATα sml1∆::URA3 tel1∆::HygMX4* | This work |
| H11 | ws1t1-10D | *MATα sml1∆::URA3 tel1∆::HygMX4* | This work |
| H12 | wm1s1-1D | *MATα mec1∆::LEU2 sml1∆::URA3* | This work |
| H13 | wm1s1-7B | *MATα mec1∆::LEU2 sml1∆::URA3* | This work |
| H14 | wm1s1-12A | *MATa mec1∆::LEU2 sml1∆::URA3* | [1] |
| H15 | wm1s1se1-3D | *MATα mec1∆::TRP1 sml1∆::HIS3 set1∆::NatMX4* | This work |
| H16 | wtH4m1s1-3D | *MATα hhf1∆::HygMX4 hhf2∆::KanMX4 (p413TARtetH4) mec1∆::LEU2 sml1∆::URA3* | This work |
| H17 | wtH4m1s1-4B | *MAT a hhf1∆::HygMX4 hhf2∆::KanMX4 (p413TARtetH4) mec1∆::LEU2 sml1∆::URA3* | This work |
| H18 | wtH4t1-1 | *MATα hhf1∆::HygMX4 hhf2∆::KanMX4 (p413TARtetH4) tel1∆::NatMX4* | This work |
| H19 | wtH4t1s1-11D | *MATα hhf1∆::HygMX4 hhf2∆::KanMX4 (p413TARtetH4) tel1∆::NatMX4 sml1∆::URA3* | This work |
| H20 | wt1s1G::R52-8A | *MATa ade2-1::ADE2 tel1∆::HygMX4 sml1∆::URA3 rad52::KanMX4::GAL1-3HA-RAD52* | This work |
| H21 | wm2m1s1-2B | *MATa rad5-535 mad2∆::KanMX4 mec1∆::LEU2 sml1∆::URA3* | This work |
| H22 | wtlc1-4B | *MATα tlc1∆::HygMX4* | This work |
| H23 | wtlc1-3A | *MATα tlc1∆::HygMX4* | This work |
| H24 | wr52-5A | *MATa rad52∆::HygMX4* | This work |
| H25 | wr51-2 | *MATa rad51∆::kanMX4 bar1∆* | This work |
| H26 | wtlc1-6 | *MATα tlc1∆::HygMX4* | This work |
| H27 | wtlc1-3 | *MATα tlc1∆::HygMX4* | This work |
| H28 | whtzs1m1-1A | *MATα htz1∆::HygMX4 sml1∆::URA3 mec1∆::LEU2* | This work |
| H29 | wm1s1se1-5A | *MATa mec1∆::TRP1 sml1∆::HIS3 set1∆::NatMX4* | This work |
| H30 | wt1s1r52-5B | *MATα tel1∆::HygMX4 sml1∆::URA3 rad52∆::HygMX4* | This work |
| H31 | w303-1aR5 | *MATa leu2-3,112 trp1-1 ura3-1 ade2-1 can1-100 his3-11,15 RAD5* | [1] |
| H32 | wc1r106m1s1-1A | *MATα cac1::Kan rtt106::Hyg mec1∆::LEU2 sml1∆::URA3* | This work |
| H33 | wc1r106m1s1-4A | *MATα cac1::Kan rtt106::Hyg mec1∆::LEU2 sml1∆::URA3* | This work |
| H34 | MKOS-3C | *MATα leu2∆::SFA trp1-1 ura3-1 ade2-1 can1-100 his3-11,15 ade3::GalHO* | [3] |
| H35 | MKOS-3C yku70 | *MATα leu2∆::SFA trp1-1 ade3::GalHO yku70∆::Hyg* | This work |
| H36 | wm1s1G::R51-2A | *MATα mec1∆::LEU2 sml1∆::URA3 TRP1::GAL1-RAD51* | This work |
| H37 | wm1s1G::R51-6D | *MATa mec1∆::LEU2 sml1∆::URA3 TRP1::GAL1-RAD51* | This work |
| H38 | WYUR17 | *ade2-1::ADE2 ARS608::HIS3 ARS609::TRP1 rad52::kanMX4::GAL1-3HA-RAD52 rad5-535* | [4] |

*All the strains are W303 background (leu2-3,112 trp1-1 ura3-1 ade2-1 can1-100 his3-11,15 RAD5), except*

*MKOS-3C* and *MKOS-3C yku70* that are related to *W303*. *Only the relevant genotypes are shown in the table.*

**References**

1. González-Prieto R, Muñoz-Cabello AM, Cabello-Lobato MJ, Prado F. Rad51 replication fork recruitment is required for DNA damage tolerance. EMBO J. 2013;32: 1307–1321. doi:10.1038/emboj.2013.73

2. Murillo-Pineda M, Cabello-Lobato MJ, Clemente-Ruiz M, Monje-Casas F, Prado F. Defective histone supply causes condensin-dependent chromatin alterations, SAC activation and chromosome decatenation impairment. Nucleic Acids Research. 2014;42: 12469–12482. doi:10.1093/nar/gku927. *Corrigendum*: Nucleic Acids Research. 2016. doi:10.1093/nar/gkw058

3. González-Barrera S. Defective nucleotide excision repair in yeast hpr1 and tho2 mutants. Nucleic Acids Research. 2002;30: 2193–2201. doi:10.1093/nar/30.10.2193

4. Vázquez MV, Rojas V, Tercero JA. Multiple pathways cooperate to facilitate DNA replication fork progression through alkylated DNA. DNA Repair. 2008;7: 1693–1704. doi:10.1016/j.dnarep.2008.06.014
