# Supplementary material for: Histone depletion prevents telomere fusions in pre-senescent cells
Source: PLoS Genet. 2018 Jun 7;14(6):e1007407. doi: 10.1371/journal.pgen.1007407 (PMC5991667; doi:10.1371/journal.pgen.1007407)
Supplement: S2 Table — (DOCX) [file pgen.1007407.s008.docx]

**S2 Table. Oligonucleotides used in this study**

| **Oligo** | **Sequence** | **Experiment** | **Ref.** |
| --- | --- | --- | --- |
| UP HHT1 HHF1 | 5´-ATGATGATGCCTCGAGGTTTAATATGTAGCTATATA-3´ | Cloning p46-H3.4.2A.2B | This work |
| LO HHT1 HHF1 | 5´-ATGATGATGCGAATTCTTTCTAAAATCCAAATACAA-3´ | Cloning p46-H3.4.2A.2B | This work |
| UP HTA1 HTB1 | 5´-ATGATGATGCGAATTCAATCGTGAGCTTTTTTCCTT-3´ | Cloning p46-H3.4.2A.2B | This work |
| LO HTA1 HTB1 | 5´-ATGATGATGCGCGGCCGCAAAATCTAGTATTGTTCACA-3´ | Cloning p46-H3.4.2A.2B | This work |
| ChrV-R-TTF | 5´-AAGAATTCGGTAAGAGACAACAGGGCTTGGAGG-3´ | T-TFs analysis | [1] |
| ChrXV-L-TTF | 5´-AAGAATTCTATGGTTAAATGGGGCAGGGTAACG-3 | T-TFs analysis | [1] |
| His4-UP | 5´-TCTGGCCTCATGGAATAGTAAGAAGGA-3 | T-TFs analysis | [1] |
| HIS4-LO | 5´- ATAAACGCCACGACCCAAATCGATG-3´ | T-TFs analysis | [1] |
| UP probe Tel. Y | 5´-TGCCGTGCAACAAACACTAAATCAA-3´ | Length telomeres probe | This work |
| LO probe Tel.Y | 5´-CGCTCGAGAAAGTTGGAGTTTTTCA-3´ | Length telomeres probe | This work |
| UP probe YCL076W | 5´-AACAGTCGCTGTACGGTATC-3´ | TEL03L Indirect end-labelling probe | [2] |
| LO probe YCL076W | 5´-TCCAAGCGAATCATCACATA-3´ | TEL03L indirect end-labelling probe | [2] |
| Y´L specific probe (UP) | 5´-GGCGTTGCAATGTGGAAATG-3´ | Y´element indirect end-labelling probe | [3] |
| Y´L specific probe (LO) | 5´-GACCGGCAAAAGCGAGTAGC-3´ | Y´element indirect end-labelling probe | [3] |
| Lo TG repeats | 5´-ACACCCACACACCACACCCAC-3 | TELOBLOT probe | This work |

**References**

1. Mieczkowski PA, Mieczkowska JO, Dominska M, Petes TD. Genetic regulation of telomere-telomere fusions in the yeast Saccharomyces cerevisae. Proc Natl Acad Sci USA. 2003;100: 10854–10859. doi:10.1073/pnas.1934561100

2. Venditti S, Vega-Palas MA, Di Mauro E. Heterochromatin Organization of a Natural Yeast Telomere. Journal of Biological Chemistry. 1999;274: 1928–1933. doi:10.1074/jbc.274.4.1928

3. Lee JY, Kozak M, Martin JD, Pennock E, Johnson FB. Evidence That a RecQ Helicase Slows Senescence by Resolving Recombining Telomeres. de Lange T, editor. Plos Biol. 2007;5: e160–11. doi:10.1371/journal.pbio.0050160
